# A parametric bootstrap control chart for Lindley Geometric percentiles

**DOI:** 10.1371/journal.pone.0316449

**Published:** 2025-02-06

**Authors:** Muthanna Ali Hussein Al-Lami, Hossein Jabbari Khamnei, Ali Akbar Heydari

**Affiliations:** 1 Department of Statistics, Faculty of Mathematics, Statistics and Computer Science, University of Tabriz, Tabriz, Iran; 2 Ministry of Education of Iraq, Baghdad, Iraq; Abdul Wali Khan University Mardan, PAKISTAN

## Abstract

Control charts are vital for quality control and process monitoring, helping businesses identify variations in production. Traditional control charts, like Shewhart charts, may not work well for skewed distributions, such as the Lindley geometric distribution (LG). This study introduces a new control chart that uses parametric bootstrap techniques to monitor percentiles of the LG distribution, providing a more effective quality control method. The LG distribution is useful for modeling material strength and failures, especially in structural design, where lower percentiles indicate reduced tensile strength. We conducted extensive simulations to assess the proposed control chart’s effectiveness, considering various distribution parameters, percentile values, Type I error rates, and sample sizes. Our findings highlight how subgroup size, percentiles, and significance levels affect control limits, stressing the need for careful parameter selection in monitoring processes. The results show that the new control chart is highly sensitive to changes in LG distribution parameters and performs consistently across different percentiles. This suggests its practical relevance and robustness for industrial applications in quality control. Future research should explore its performance in real-world production settings to confirm its efficiency and reliability.

## 1. Introduction

The standard procedures of statistical quality control (SQC) often rely on control charts and acceptance sampling plans, which are typically based on the assumption of normal data. However, in practice, this assumption rarely holds true. Upon analyzing various data sets from different applications, such as statistical process control (SPC), we have observed that this type of data often displays moderate to strong asymmetry and light to heavy tails. Therefore, fitting a normal distribution to the data is generally not the most suitable option. Additionally, modeling real data sets, even when potential symmetric models for the underlying data distribution exist, is always challenging due to uncontrollable perturbation factors.

To address this issue, it is necessary to use distributions with more flexible characteristics as the quality characteristic distribution. The introduction of new distributions is driven by the goal of transitioning from former distributions to more flexible structures. The number of parameters, as well as the method of defining the distribution and its structure, are crucial for fitting and flexibility. An ideal distribution should exhibit better fitting, be more flexible in shape, and have easier formulas for implementation. The Lindley distribution [1] is one of the distributions particularly suitable for studying the reliability modeling of stress-strength characteristics due to its excellent properties. Hence, many researchers have proposed new classes of distributions based on extensions and modifications of the Lindley distribution. Table 1 shows a literature review of the distributions introduced based on the Lindley distribution, along with some of their most important applications, including reliability and SPC fields.

**Table 1:** Review of literature about the distributions introduced based on the Lindley distribution.

Article	Presents or introduces	Results
[2]	The Kumaraswamy Lindley distribution and investigates its properties and applications.	The statistical characteristics of this distribution and its potential use in modeling various phenomena.
[3]	The properties of this distribution and presents its application in mathematical and computational simulations.	A comprehensive analysis and demonstrate the usefulness of this distribution in various scenarios.
[4]	Explore the characteristics and applications of this statistical distribution through mathematical and computational simulations.	Contributes to the understanding and application of the Lindley distribution in diverse contexts.
[5]	Delves into the Generalized Lindley distribution and presents relevant mathematical formulations and analyses.	The properties and applications of this distribution, expanding the literature on the subject.
[6]	Propose a generalized version of the Poisson-Lindley distribution.	Investigate its properties, including moments and estimation methods.
[7]	A method for generating random variables with the Lindley or Poisson-Lindley distribution using the Lambert W function.	An efficient tool for simulating data with these distributions.
[8]	Introduce the negative binomial-Lindley distribution and explore its practical applications.	The distribution’s properties and provide examples of its usage in various fields.
[9]	Reliability estimation in Lindley distribution with progressively type II right censored sample.	Methods for parameter estimation and conduct simulations to evaluate the performance of the proposed methods.
[10]	Introduce a two-parameter weighted Lindley distribution and explore its applications in analyzing survival data.	The estimation of the distribution’s parameters and present an application example using real survival data.
[11]	The use of the Lindley distribution in analyzing competing risks lifetime data.	The estimation of the distribution parameters and apply the Lindley model to real data to demonstrate its usefulness in modeling competing risks.
[12]	An extension to the Lindley distribution.	The distribution’s properties and discuss its applicability in statistical modeling.
[13]	A new lifetime distribution with two parameters.	The model and its properties, offering insights into its potential use in various applications.
[14]	Examines the mean residual life function and stress and strength analysis for the Lindley distribution.	Discusses various loss functions and their impact on the analysis.
[15]	Focuses on the reliability analysis of the Lindley distribution, specifically considering the presence of an outlier.	Insights and findings provided are limited in scope, they offer a valuable perspective for researchers interested in reliability modeling.
[16]	The transmuted Lindley-geometric distribution and explores its applications.	Insights provide opportunities for additional research and practical applications.
[17]	Examine the moments, hazard function, and reliability properties of the beta-Lindley distribution to illuminate its potential utility in various fields.	A basis for additional research and practical applications.
[18]	Discussing about the Lambert W package, which is a computational tool used for evaluating.	Provide information on the features and capabilities of the package.
[19]	Focus on the Lindley-Exponential distribution and explore its properties and applications.	Analysis of the distribution and highlight its potential uses in various fields.
[20]	Introducing the Quasi-Lindley Geometric distribution and analyze its statistical properties.	Investigate the distribution’s moments, generating function, probability mass function, and other important characteristics.
[21]	A generalized class of the Kumaraswamy Lindley distribution and investigates its applications in analyzing lifetime data.	Providing a detailed exposition of the properties and characteristics of this distribution.
[22]	A new class of generalized Lindley distributions and examine its practical applications.	Analyzing of the proposed distribution, including its moments, hazard rate, and stochastic ordering properties.
[23]	Focusing on the weighted Lindley distributions and their implications for inferences on stress-strength reliability.	The properties of these distributions and their potential applications in reliability analysis, offering insights into statistical methodologies for modeling reliability problems.
[24]	Introducing the odd Lindley-G family of distributions.	Exploring the statistical properties and applications of this family.
[25]	A new generalized Poisson Lindley distribution.	The distribution’s statistical properties, such as moments, moment-generating function, quantile function, and order statistics.
[26]	A new distribution called the generalized beta-generated Lindley distribution for describing remission times.	The distribution’s mathematical properties, including its moments, probability density function, hazard function, and quantile function.
[27]	A new statistical distribution called the Ristić and Balakrishnan Lindley-Poisson Distribution.	A comprehensive overview of the distribution, including its mathematical properties and theoretical foundations.
[28]	A new statistical distribution called the Exponentiated Lindley Geometric Distribution.	Exploring the distribution’s properties and derive several key statistical measures.
[29]	A new extension of the Lindley Geometric Distribution.	Providing a thorough explanation of the extended distribution’s properties, including its moments and probability functions.
[30]	Exploring the properties and applications of the binomial mixture Lindley distribution.	Investigating the characteristics and behavior of this distribution, potentially including aspects such as moments, cumulative distribution function, and other statistical properties.
[31]	Investigating the Lindley negative-binomial distribution and its properties, estimation methods, and applications in analyzing lifetime data.	The statistical characteristics of this distribution, methodologies for estimating its parameters, and how it can be applied in modeling and analyzing lifetime or survival data.
[32]	focusing on Lindley power series distributions.	exploring the properties and characteristics of these distributions, including moments, probability density function, cumulative distribution function, and other statistical properties.
[33]	Introducing the Power Lindley Geometric Distribution as a novel model for analyzing failure in business.	Exploring the statistical properties and applications of this distribution in the context of failure analysis.
[34]	The estimation of parameters for the Lindley-Geometric distribution.	Provide a detailed analysis of the distribution and propose a method for estimating its parameters.
[35]	Propose a new class of bivariate Lindley distributions derived from stress and shock models.	Focusing on exploring the reliability properties of these distributions.
[36]	examining various characteristics and properties of the Poisson-Lindley distribution, such as moments, hazard rate, stochastic ordering, and reliability.	Exploring the application of this distribution in statistical process control (SPC).

Recently, by compounding Lindley and geometric distributions, [13] introduced the Lindley-geometric (LG) distribution. Advantages of using the Lindley-Geometric distribution include:

1. Flexibility: The *LG* distribution offers flexibility in modeling continuous data with additional parameters, allowing for a better fit to the data and capturing various patterns and characteristics;
2. Overdispersion and under dispersion: The *LG* distribution can effectively model data exhibiting overdispersion or under dispersion, providing a more accurate representation of the variance in the data compared to other distributions;
3. Versatility: The *LG* distribution can be applied in various fields such as finance, economics, and environmental studies, making it a versatile tool for modeling continuous data;
4. Statistical inference: The *LG* distribution allows for improved statistical inference and analysis of continuous data, enabling researchers to make more precise predictions and draw meaningful conclusions from the data.

Therefore, The Lindley geometric distribution can be utilized in statistical process control methodologies to monitor and improve quality control processes by providing a robust framework for analyzing and interpreting quality control data. So, in this article, we will present control charts for the quality characteristics that follow the *LG* distribution.

Various researchers have explored the use of bootstrap techniques in statistical process control charts. The non-parametric bootstrap approach can be utilized in control charts, removing the need for conventional parametric assumptions. The bootstrap techniques can also be applied in cases where the distribution of the statistic used for process monitoring is unknown. [37] introduced a bootstrap control chart for monitoring the process mean, offering an alternative to Shewhart’s $\bar{X}$ chart, particularly useful for non-normal process data. Researchers such as [38–40] established the groundwork by introducing bootstrap control charts for quality engineering, process control, and reliability analysis. These investigations underscored the advantages of employing bootstrap methods to develop customized control charts tailored to distinct distributions and process parameters. Subsequent research led by [41–50] further advanced the use of bootstrap methods in developing control charts for various distributions and applications in quality control. These studies provided valuable insights into the reliability, efficiency, and novel approaches to control charting using bootstrap resampling techniques.

Innovative strategies introduced by researchers like [51–56] aimed to enhance the performance and accuracy of control charts. These approaches included Hotelling’s *T*^2^ control charts, model selection methods, and adjustments to control limits, with a focus on improving the detection of out-of-control conditions and upholding quality standards in industrial processes.

Recent studies in the 2020s conducted by researchers such as [36, 57–63] have delved into new frontiers in bootstrap control charting. These studies explore areas such as monitoring non-normal processes, analyzing the availability index, handling proportion data, and examining specific distribution percentiles. They provide valuable insights into overcoming challenges in quality control, manufacturing systems, and reliability data analysis through the application of bootstrap techniques.

The focus of this study lies in the *LG* distribution, which has been applied in modeling material strength and various types of failures, as demonstrated in studies such as [13, 34].

There is no explicit formula or known sampling distribution of a statistic for the *LGD* percentile in cases of small sample sizes. Therefore, in this research, we introduce a new control chart using parametric bootstrap for *LGD* percentiles.

This paper is organized as follows: We provide some of the characteristics of the *LGD* in Section 2. The procedure of a parametric bootstrap control chart for *LGD* percentiles is presented in Section 3. The performance of the bootstrap Lindley-geometric percentile control chart is examined in Section 4. Illustrative examples in a simulated data set and in a real-world data set are included in Section 5. The paper ends with discussions and concluding remarks in Section 6.

## 2. Some of the characteristics of the *LGD*

In this section, we will first explain some characteristics of the *LG* distribution, such as the graph of the probability distribution function(pdf), in addition to the mean, variance, and quantile function. Then, we will talk about estimating the parameters, and generating random numbers of the *LGD*.

[13] introduced the *LG* distribution with pdf and cumulative distribution function (cdf) given by

$$f_{LG}(y,\theta ,p)=\frac{\theta^{2}}{\theta +1}(1-p)(1+y)e^{-\theta y}{\left[1-p\left(1+\frac{\theta y}{\theta +1}\right)e^{-\theta y}\right]}^{-2};\;y>0,\;\theta >0,\;0<p<1,$$ (1)

$$F_{LG}(y,\theta ,p)=\frac{1-\left(1+\frac{\theta y}{\theta +1}\right)e^{-\theta y}}{1-p\left(1+\frac{\theta y}{\theta +1}\right)e^{-\theta y}};\;y>0,\;\theta >0,\;0<p<1.$$ (2)

**Fig 1:**
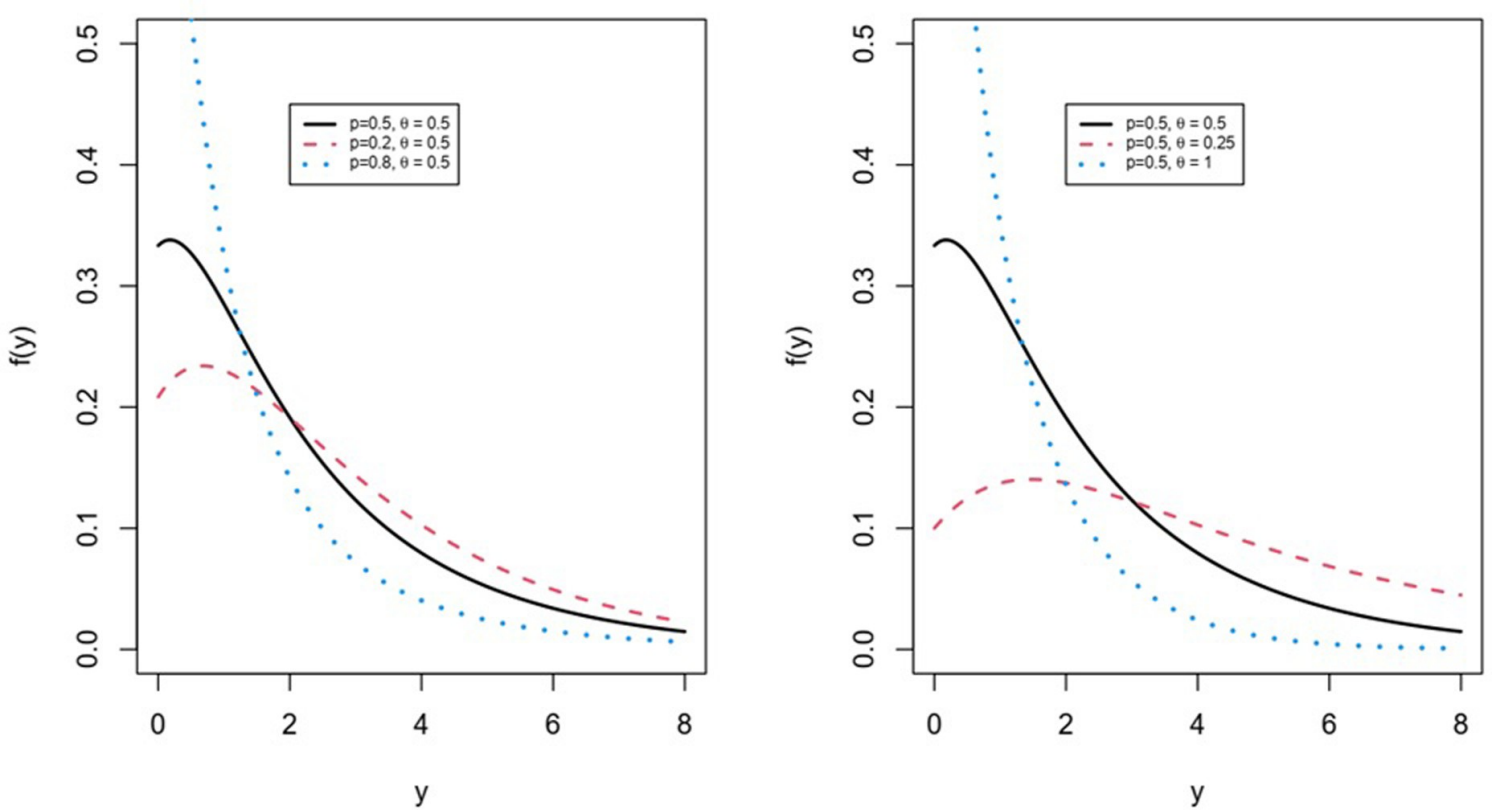
pdf of *LG* distribution for different values of *θ* and *p*.

Several distinct sub-models of the *LG* distribution (1) can be derived. For the Lindley distribution, the case where *p* = 0 is considered. As *p* approaches from left to 1, the *LG* distribution converges to a degenerate distribution at zero. Consequently, the parameter *p* is regarded as a concentration parameter. The density function of the *LG* distribution exhibits the following characteristics: (i) it decreases for all combinations of *p* and *θ* where p is greater than (1-*θ*^2^)/(1 + *θ*^2^), and (ii) it is unimodal for all combinations of *p* and θ where *p* is less than or equal to (1-*θ*^2^)/(1 + *θ*^2^). Fig 1 illustrates the *pdf* for the *LGD* in Eq (1), showing how it changes at different values of *θ* and *p*.

### 2.1 Moments of the *LGD*

Assuming *Y* follows a *LG*(*p*, *θ*) distribution, utilizing Eq (1) and applying the binomial expression for ${\left(1+\frac{\theta y}{\theta +1}\right)}^{j}$, the *r—th* moment of *Y* can be expressed as

$$E\left(Y^{r}\right)=\frac{\theta^{2}(1-p)}{\theta +1}\sum_{j=0}^{\infty }\sum_{i=0}^{j}\left(\begin{array}{c} j\\ i \end{array}\right)(j+1)p^{j}{\left(\frac{\theta }{\theta +1}\right)}^{i}\frac{\Gamma (r+i+1)}{{\left(\theta (j+1)\right)}^{r+i+1}}\left(1+\frac{r+i+1}{\theta (j+1)}\right).$$

Replacing the *r* = 1 in the above equation, yields the mean of the *LGD* as

$$E(Y)=\frac{\theta^{2}(1-p)}{\theta +1}\sum_{j=0}^{\infty }\sum_{i=0}^{j}\frac{(j+1)!}{(j-i)!}p^{j}{\left(\frac{\theta }{\theta +1}\right)}^{i}\frac{i+1}{{\left(\theta (j+1)\right)}^{i+2}}\left(1+\frac{i+2}{\theta (j+1)}\right).$$

Also, the variance of *LGD* is obtained by substituting *r* = 2 in the moment expression and using the following relationship

$$Var(Y)=E\left(Y^{2}\right)-{\left(E(Y)\right)}^{2}.$$

### 2.2 Quantile function of the *LGD*

Let *Y* be an arbitrary random variable with *cdf F*(*y*) = *Pr*(*Y* ≤ *y*), where *x* ∈ *R*. For any *u* ∈ (0,1), the *u*—*th* quantile function, *Q*(*u*) of *Y* is the solution of

*F* (*Q*(*u*)) = *u*,

for *Q*(*u*) > 0.

For any fixed *θ* > 0, from Eq (2), we obtain

$$\frac{1-\left(1+\frac{\theta Q(u)}{\theta +1}\right)e^{-\theta Q(u)}}{1-p\left(1+\frac{\theta Q(u)}{\theta +1}\right)e^{-\theta Q(u)}}=u$$

$$-\frac{1-u}{1-pu}=-\left(\frac{\theta +1+\theta Q(u)}{\theta +1}\right)e^{-\theta Q(u)}$$

$$-\frac{(u-1)(\theta +1)e^{-(\theta +1)}}{pu-1}=-(\theta Q(u)+\theta +1)e^{-(\theta Q(u)+\theta +1)}$$

In the above equation, we note that (*θQ*(*u*)+*θ* + 1) is the Lambert *W* function of the real argument $\frac{u-1}{up-1}(\theta +1)e^{-(\theta +1)}.$ The Lambert *W* function is defined by

$$W(x)e^{W(x)}=x.$$

The Lambert function has two real branches with a branching point located at (-*e*^-1^,1). The lower branch, *W*_-1_(*y*), is defined in the interval [-*e*^-1^,1] and has a negative singularity for *x* → 0^-^. The upper branch, *W*_0_ (*y*), is defined for *y* ∈ [-*e*^-1^,∞].

Then, we have

$$W\left(-\frac{(u-1)(\theta +1)e^{-(\theta +1)}}{pu-1}\right)=-(\theta Q(u)+\theta +1)$$ (3)

Clearly, for any *θ* > 0, 0 < *p* < 1 and u ∈(0,1), we have (*θQ*(*u*)+*θ*+1) > 1 and then $-\frac{u-1}{up-1}(\theta +1)e^{-(\theta +1)}<0$. Therefore, considering the lower branch of the Lambert *W* function, we can write Eq (3) as

$$W_{-1}\left(-\frac{(u-1)(\theta +1)e^{-(\theta +1)}}{pu-1}\right)=-(\theta Q(u)+\theta +1).$$

Hence, the quantile function of *Y* is given by

$$Q(u)=-\frac{W_{-1}\left(-\frac{(u-1)(\theta +1)e^{-(\theta +1)}}{pu-1}\right)+\theta +1}{\theta }.$$ (4)

### 2.3 Estimation of the parameters of the *LGD*

It is necessary to estimate the parameters as accurately as possible to obtain efficient information about the distribution. The maximum likelihood method is generally preferred for parameter estimation.

We consider the maximum likelihood estimation (*MLE*) about the parameters (*θ*,*p*) of the *LGD*. Suppose *y*_*obs*_ = {*x*_1_,*x*_2_,…,*x*_*n*_} is a random sample of size n from the *LGD*. Then the log-likelihood function is given by

$$\begin{array}{l} l=log\prod_{i=1}^{n}f\left(x_{i}\right)\\ =2n\;log(\theta )-n\;log(\theta +1)+n\;log(1-p)+\sum_{i=1}^{n}log\left(1+x_{i}\right)-\theta \sum_{i=1}^{n}x_{1}\\ -2\sum_{i=1}^{n}log\left[1-p\left(1+\frac{\theta x_{i}}{\theta +1}\right)e^{-\theta x_{i}}\right]. \end{array}$$ (5)

The *MLE*s of *p* and *θ* are obtained by solving the following equations:

$$\frac{\partial l}{\partial \theta }=\frac{2n}{\theta }-\frac{n}{\theta +1}-\sum_{i=1}^{n}x_{i}-2p\sum_{i=1}^{n}\frac{x_{i}e^{-\theta x_{i}}\left[\left(1+\frac{\theta x_{i}}{\theta +1}-\frac{1}{{\left(\theta +1\right)}^{2}}\right)\right]}{{\left[1-p\left(1+\frac{\theta x_{i}}{\theta +1}\right)e^{-\theta x_{i}}\right]}^{2}}=0,$$ (6)

$$\frac{\partial l}{\partial p}=-\frac{n}{1-p}+2\sum_{i=1}^{n}\frac{\left(1+\frac{\theta x_{i}}{\theta +1}\right)e^{-\theta x_{i}}}{1-p\left(1+\frac{\theta x_{i}}{\theta +1}\right)e^{-\theta x_{i}}}=0.$$ (7)

However, they do not lead to explicit analytical solutions for the parameters. Thus, the estimates can be obtained by means of numerical procedures such as Newton-Raphson method. The *R* software provides the nonlinear optimization routine for solving such problems. Also, to obtain the *MLE* of the *LGD*, [13] introduced an *EM* Algorithm.

The other methods of estimation methods of the *LGD* parameters such as least-squares, weighted least-squares, Anderson-Darling, and Crámer–von-Mises are presented by [34].

### 2.4 Generating random numbers from *LGD*

In this section, we discuss how to generate random numbers from the *LGD*. One reliable method for generating random numbers for the *LGD* is using the quantile function for the distribution. Instead of determining the cumulative probabilities for a set of values, the quantile function determines the values for a set of cumulative probabilities. By utilizing the quantile function, we can generate random numbers from a uniform distribution and then transform them into the distribution of interest using its quantile function.

So, we can generate a random number *x* from the *LGD* using the following algorithm.

### Algorithm 1: Generating a random number from the *LGD*

Step 1: Generate *u* from a *Uniform* (0,1) distribution.
Step 2: calculate *x* = *Q*(*u*) where *Q*(*u*) is calculated from Eq (4).

## 3. Bootstrap control charts for percentiles of the *LGD*

Understanding the strength distribution is crucial for structural design purposes, especially when considering the lower percentiles that signify a decrease in material tensile strength. Given the asymmetric nature of the *LG* distribution, traditional $\bar{X}$ and *R* Shewhart control charts may not effectively detect shifts in specific lower percentiles, particularly for quality characteristics like the breaking strength of brittle materials. Our focus is on utilizing the parametric bootstrap (percentile) method to develop control charts for *LG* distribution.

The following algorithm, which is similar to those proposed by [41] can be used to construct the bootstrap Lindley Geometric percentile control chart.

### Algorithm 2: Constructing the bootstrap percentile control chart for the *LGD*

1. Gather n × m observations from an in-control, stable process assuming they follow a *LGD* with unknown parameters *p* and *θ*. The observations, denoted as *x*_*ij*_ with *i* = 1, …,*n* and *j* = 1, …,*m*, are drawn from *m* independent subgroups of size *n*.
2. Determine the maximum likelihood estimators (*MLE*s), $\hat{p}$ and $\hat{\theta }$, for the unknown parameters using all *n* × *m* observations. These estimators are obtained by solving the Eqs (6) and (7).
3. Create a parametric bootstrap subgroup sample of size $n,x_{1}^{*},x_{2}^{*},\ldots ,x_{n}^{*}$, from the *LGD* using the maximum likelihood estimators, $\hat{p}$and $\hat{\theta }$ which are calculated from step 2, as the estimated parameters.
4. Determine the maximum likelihood estimators from the bootstrap subgroup sample and represent them as ${\hat{p}}^{*}$ and ${\hat{\theta }}^{*}$.
5. For the bootstrap subgroup sample, calculate $Q^{*}(u)=\frac{W_{-1}\left(-\frac{(u-1)\left({\hat{\theta }}^{*}+1\right)e^{-\left({\hat{\theta }}^{*}+1\right)}}{{\hat{p}}^{*}u-1}\right)+{\hat{\theta }}^{*}+1}{{\hat{\theta }}^{*}}$, which is the estimated 100*u*-th percentile, *Q*(*u*).
6. Iterate through steps 3–5 numerous times, denoted as *B*, to acquire *B* bootstrap estimates of *Q*(*u*), labeled as $Q_{1}^{*}(u),Q_{2}^{*}(u),\ldots ,Q_{B}^{*}(u).$.
7. Sort the *B* bootstrap estimates $Q_{i}^{*}(u)$ in ascending order. The *LCL* is the value of the smallest ordered $Q_{i}^{*}(u)$ that has (*α*/2)*B* values below it. In this context, *α* represents the probability of considering an observation as out of control when the process is actually in control (commonly set at 0.0027 for Shewhart-type charts). The *UCL* is the value of the smallest ordered $Q_{i}^{*}(u)$ that has (*α*/2)*B* values above it.

After calculating the control limits, for implementation the phase II of the bootstrap control chart, subsequent subgroup samples of size *n* are collected from the process at regular intervals, and *Q*(u) is estimated for each new subgroup using the MLE as described in step 5. If the estimate, $\hat{Q}(u)$, falls within the UCL and LCL determined in step 7, the process is considered to be in control. Any $\hat{Q}(u)$ values below the *LCL* or above the *UCL* suggest process may be out of control. Therefore, following the determination of bootstrap control limits, the process is monitored using the statistic $\hat{Q}(u)$ in the conventional manner.

The subsequent section will evaluate the effectiveness of the suggested bootstrap Lindley-geometric percentile control chart through computer simulations. Specifically, an examination of the statistical properties of the bootstrap Lindley-geometric percentile control limits will be conducted. Additional simulations will assess the performance of the bootstrap percentile control chart in terms of Average Run Lengths (*ARL*s), and their standard deviations (*SDARL*s).

## 4. Performance of the bootstrap Lindley-geometric percentile control chart

This section includes computer simulations that assess the effectiveness of the bootstrap Lindley-geometric percentile control chart. The analysis involves investigating the behavior of the bootstrap control limits by determining the average *UCL* and *LCL* (*MLCL*, and *MUCL*) along with their respective standard deviations (*SDLCL*, and *SDUCL*) from the simulations. Additionally, further simulations are conducted to evaluate the *ARL* when the process is in control, along with the associated standard deviation for each *ARL* (i.e., *SDRL*). These simulations encompass diverse sample sizes, various percentiles of interest, and different levels of *α*. The *ARL* is also computed for when the process is out of control, accompanied by its variance. Similar simulations are carried out with varying sample sizes, different percentiles of interest, and various *α* values.

The mean *UCL* and *LCL* along with their respective standard deviations were calculated as follows: A total of *n* × 25 observations were generated from a *LG* distribution with parameters *p* and *θ*, following the Algorithm 1, and the procedure outlined in step 1 of Algorithm 2. Subsequently, steps 2 to 7 of Algorithm 2 were executed. The value of *B* was set to 10,000 in this scenario. This entire sequence of steps (1–7) was iterated *k* = 100 times, and the mean *LCL* and mean *UCL* were determined from the 100 data sets created using the Monte Carlo method. Additionally, the standard deviations of the control limits were calculated based on these 100 values. The simulation process was conducted across various sample sizes (*n* = 4,5,6), different percentiles (*u* = 0.05,0.10), different parameters of the *LG* distribution (*θ* and *p*), and diverse *α* values (*α* = 0.0027,0.002,0.01). The simulations were implemented using the R software, and Tables 2–4 present some of the outcomes. As anticipated, an increase in subgroup size from four to five to six leads to the convergence of control limits, while elevating the percentile from 0.05 to 0.10 results in the widening of the limits, and lower *α* (significance level) leads to wider control limits. Additionally, an increase in any of the *θ* or *p* parameters of the *LG* distribution narrows the control limits.

**Table 2:** Average control limits along with their respective standard deviations for *α* = 0.0027.

*n*	*LGD* Parameters		Percentile of interest							
			*u* = 0.05				*u* = 0.1			
	*θ*	*P*	*MLCL*	*SDLCL*	*MUCL*	*SDUCL*	*MLCL*	*SDLCL*	*MUCL*	*SDUCL*
4	0.25	0.25	0.045210	0.004337	2.296595	0.070377	0.093523	0.009045	3.818743	0.104305
	0.25	0.75	0.007209	0.000636	0.504328	0.018180	0.015730	0.001438	0.939527	0.029788
	0.75	0.25	0.015628	0.001533	1.532578	0.069161	0.032640	0.003744	2.610101	0.108725
	0.75	0.75	0.002555	0.000326	0.317525	0.010812	0.005158	0.000508	0.600041	0.025635
5	0.25	0.25	0.063083	0.005797	2.079311	0.053178	0.130892	0.010912	3.448046	0.085876
	0.25	0.75	0.010369	0.000869	0.449215	0.013764	0.022129	0.002247	0.834353	0.021595
	0.75	0.25	0.022222	0.001441	1.324147	0.048335	0.046720	0.004040	2.277760	0.070056
	0.75	0.75	0.003517	0.000369	0.267558	0.009071	0.007343	0.000722	0.522534	0.016653
6	0.25	0.25	0.081092	0.005827	1.926460	0.041702	0.165310	0.013393	3.210210	0.065613
	0.25	0.75	0.013149	0.000902	0.409196	0.011708	0.027268	0.001348	0.767854	0.020470
	0.75	0.25	0.028350	0.001854	1.198950	0.041818	0.058784	0.003633	2.047049	0.081526
	0.75	0.75	0.004560	0.000408	0.237442	0.008605	0.009507	0.000527	0.460416	0.019287

**Table 3:** Average control limits along with their respective standard deviations for *α* = 0.002.

*n*	*LGD* Parameters		Percentile of interest							
			*u* = 0.05				*u* = 0.1			
	*θ*	*p*	*MLCL*	*SDLCL*	*MUCL*	*SDUCL*	*MLCL*	*SDLCL*	*MUCL*	*SDUCL*
4	0.25	0.25	0.042191	0.004866	2.373942	0.089102	0.087452	0.009865	3.910622	0.108922
	0.25	0.75	0.006469	0.001022	0.533665	0.019791	0.014249	0.001521	0.980840	0.036147
	0.75	0.25	0.014536	0.001295	1.592585	0.055797	0.030037	0.003666	2.733700	0.098387
	0.75	0.75	0.002212	0.000249	0.332600	0.016246	0.004592	0.000472	0.633416	0.027838
5	0.25	0.25	0.057719	0.004772	2.135123	0.056216	0.124736	0.011453	3.550046	0.102806
	0.25	0.75	0.009389	0.001087	0.469270	0.014387	0.019975	0.002168	0.875487	0.022303
	0.75	0.25	0.020954	0.001645	1.418256	0.081716	0.042666	0.004347	2.368461	0.085041
	0.75	0.75	0.003132	0.000345	0.282841	0.013861	0.006950	0.000598	0.553585	0.031360
6	0.25	0.25	0.075978	0.006262	1.983412	0.057085	0.155064	0.012457	3.283225	0.087817
	0.25	0.75	0.011960	0.001269	0.427100	0.012611	0.025908	0.002459	0.799690	0.027458
	0.75	0.25	0.026271	0.001981	1.238341	0.049368	0.054129	0.004201	2.140220	0.076132
	0.75	0.75	0.004185	0.000399	0.248147	0.010427	0.008720	0.000831	0.482040	0.020098

**Table 4:** Average control limits along with their respective standard deviations for *α* = 0.01.

*n*	*LGD* Parameters		Percentile of interest							
			*u* = 0.05				*u* = 0.1			
	*θ*	*p*	*MLCL*	*SDLCL*	*MUCL*	*SDUCL*	*MLCL*	*SDLCL*	*MUCL*	*SDUCL*
4	0.25	0.25	0.073973	0.003980	1.979311	0.033161	0.152841	0.005510	3.303852	0.056454
	0.25	0.75	0.012372	0.000686	0.425201	0.007315	0.025746	0.000958	0.800051	0.018961
	0.75	0.25	0.026325	0.001089	1.255359	0.027965	0.054944	0.002125	2.141901	0.042464
	0.75	0.75	0.004210	0.000239	0.248700	0.005966	0.009060	0.000414	0.486634	0.013844
5	0.25	0.25	0.098416	0.004153	1.807802	0.025638	0.194888	0.009102	3.039827	0.047153
	0.25	0.75	0.016147	0.000680	0.380732	0.006126	0.033482	0.001508	0.720615	0.015443
	0.75	0.25	0.034791	0.001581	1.095889	0.020769	0.070748	0.003033	1.903249	0.035597
	0.75	0.75	0.005471	0.000251	0.218774	0.004800	0.011497	0.000468	0.424154	0.010168
6	0.25	0.25	0.118003	0.004252	1.674476	0.029928	0.234008	0.008288	2.820237	0.036343
	0.25	0.75	0.019372	0.000763	0.351380	0.007014	0.040238	0.001528	0.665543	0.012637
	0.75	0.25	0.041125	0.001418	0.991090	0.026436	0.085689	0.003201	1.744528	0.039977
	0.75	0.75	0.006627	0.000254	0.194190	0.004876	0.014144	0.000544	0.377283	0.006814

The in-control *ARL* was determined by creating *m* = 25 subgroups, each comprising *n* observations, and calculating the corresponding bootstrap *UCL* and *LCL* using steps 2–7 detailed in Algorithm 2. After establishing the control limits, forthcoming subgroup samples were generated from a *LG* distribution with identical parameters. For every new subgroup, the *MLE* was used to estimate *Q*(*u*). The count of instances where the estimate, $\hat{Q}(u)$, fell within the limits was tracked until an estimate exceeded the control limits. The run length was computed as the total instances the estimate remained within the limits plus the initial subgroup indicating an out-of-control situation. The process of determining the run length was repeated 1000 times, and the average of these 1000 run lengths (Average Run Length, *ARL*) along with their standard errors (*SDRL*) were computed. These simulations for the in-control *ARL* were conducted across various values of the parameters for decreasing and unimodal *LG* densities. The simulations were also carried out for different percentiles, with different values of *α* across all runs. To determine the out-of-control *ARL*s and related *SDRL*s, 25 sets of samples with a size of *n* were generated from a *LG* distribution with parameters *θ* and *p*. The *UCL* and *LCL* were calculated in a similar manner as for the in-control *ARL*s. Subsequent subgroup samples, each consisting of *n* observations, were then simulated from a *LG* distribution with distinct values of *θ* and *p*. The alteration in the parameters mimics a shift in the process percentile, signaling an ’out-of-control’ state to be identified. The percentile estimate, $\hat{Q}(u)$, was calculated for every subgroup of size *n*. The count of subgroups where $\hat{Q}(u)$ lies within the control limits was tallied until an estimate exceeded the limits. The run length was determined by counting the instances where the estimates stayed within the limits, along with the first subgroup indicating an out-of-control situation. This process was iterated 1000 times, and the *ARL*s and their corresponding standard errors were derived from the 1000 generated run lengths.

Selected results for the *LGD* parameters of *p* = (0.25,0.75) and *θ* = (0.25,0.75), *α* values of 0.0027, 0.002, and 0.01, and *u* values of 0.05 and 0.10 for the *ARL* simulations are presented in Tables 5–7. According to established theory, the reciprocal of the *α* value is anticipated to represent the theoretical in-control *ARL*. Given *α* = 0.0027, the in-control *ARL*s for these simulations is expected to be approximately 1/*α*, which equals 370. In Table 5, it can be seen that when the values of *θ* and *p* in the *LG* distribution do not change (i.e. in-control *ARL*s is calculated), these values are around 370. Furthermore, Tables 6 and 7 show that the in-control *ARL* values for *α* values of 0.002 and 0.01 are around 500 and 100 respectively. When the process is in-control, a smaller *ARL* suggests that the calculated control limits may be overly restrictive, while *ARL*s exceeding 1/*α* indicate that the control limits may be too lenient or that the bootstrap control charts result in fewer false alarms. For the out-of-control *ARL* simulations, the bootstrap *LGD* percentile control chart detects the shift towards an out-of-control state. In this case, the smaller the calculated value of the ARL, the quicker the control chart detects changes in the process. For example, examining the first row of Table 5 reveals that when *α* = 0.0027 and *n* = 4, for *u* = 0.05, as the parameters of the *LG* distribution shift from (*θ* = 0.25,*p* = 0.25) to (*θ* = 0.25,*p* = 0.75), the bootstrap percentile control chart detects this change on average after 8.393 sampling times (with a standard deviation of 7.764). For *u* = 0.1, this change is detected on average after 7.603 sampling times with an approximate standard deviation of 6.876.

**Table 5:** *ARL*s along with their respective standard deviations for *α* = 0.0027.

*n*	*LGD* Parameters	Percentile of interest								
			*u* = 0.05				*u* = 0.1			
	(*θ*,*p*) ⇓	(*θ*,*p*) ⇒	(0.25, 0.25)	(0.25, 0.75)	(0.75, 0.25)	(0.75, 0.75)	(0.25, 0.25)	(0.25, 0.75)	(0.75, 0.25)	(0.75, 0.75)
4	(0.25,0.25)	*ARL*	367.997	8.393	46.654	1.910	389.065	7.603	46.644	1.884
		*SDRL*	353.028	7.764	44.676	1.291	401.007	6.876	50.657	1.192
	(0.25,0.75)	*ARL*	1.687	385.688	7.165	51.573	1.737	359.233	7.349	47.342
		*SDRL*	1.065	370.983	6.531	50.608	1.127	346.541	6.459	47.546
	(0.75,0.25)	*ARL*	39.482	105.939	367.987	9.551	37.724	102.231	359.408	8.881
		*SDRL*	38.817	101.777	362.097	9.185	37.683	102.330	381.989	8.499
	(0.75,0.75)	*ARL*	1.225	39.318	2.809	360.318	1.234	36.997	2.832	377.186
		*SDRL*	0.522	39.298	2.236	379.303	0.529	35.621	2.314	384.972
5	(0.25,0.25)	*ARL*	379.379	4.928	33.015	1.388	368.619	5.049	31.074	1.398
		*SDRL*	379.710	4.336	33.876	0.717	355.340	4.569	30.044	0.742
	(0.25,0.75)	*ARL*	1.473	366.568	5.880	34.028	1.482	339.163	5.730	30.878
		*SDRL*	0.805	361.501	5.231	33.668	0.855	336.767	5.359	27.388
	(0.75,0.25)	*ARL*	24.659	74.917	382.006	5.660	24.708	73.551	349.258	5.529
		*SDRL*	22.806	75.625	378.728	5.526	25.468	70.417	351.505	4.886
	(0.75,0.75)	*ARL*	1.128	24.610	2.292	365.763	1.126	25.964	2.308	391.546
		*SDRL*	0.390	24.003	1.738	375.605	0.380	26.045	1.748	398.735
6	(0.25,0.25)	*ARL*	403.094	3.154	20.837	1.204	391.423	3.035	2.126	1.131
		*SDRL*	385.177	2.584	19.578	0.503	375.334	2.667	1.513	0.379
	(0.25,0.75)	*ARL*	1.322	362.550	5.274	24.159	1.331	371.534	5.161	24.330
		*SDRL*	0.617	373.022	4.512	23.465	0.714	384.213	4.537	24.253
	(0.75,0.25)	*ARL*	20.037	54.197	402.830	4.073	18.716	55.128	359.727	3.663
		*SDRL*	19.398	52.029	406.625	3.581	17.931	54.562	362.481	2.961
	(0.75,0.75)	*ARL*	1.057	19.897	1.924	372.032	1.052	19.412	1.847	364.553
		*SDRL*	0.249	20.349	1.331	366.407	0.231	18.168	1.275	358.410

**Table 6:** *ARL*s along with their respective standard deviations for *α* = 0.002.

*n*	*LGD* Parameters	Percentile of interest								
			*u* = 0.05				*u* = 0.1			
	(*θ*,*p*) ⇓	(*θ*,*p*) ⇒	(0.25, 0.25)	(0.25, 0.75)	(0.75, 0.25)	(0.75, 0.75)	(0.25, 0.25)	(0.25, 0.75)	(0.75, 0.25)	(0.75, 0.75)
4	(0.25,0.25)	*ARL*	464.870	9.420	58.295	2.008	446.262	9.293	55.099	2.032
		*SDRL*	494.860	8.658	55.931	1.438	439.752	8.885	55.196	1.430
	(0.25,0.75)	*ARL*	1.893	548.341	7.408	68.592	1.883	515.737	7.667	60.212
		*SDRL*	1.319	519.107	6.919	65.060	1.279	517.687	7.492	59.498
	(0.75,0.25)	*ARL*	47.923	123.323	477.266	10.935	50.653	136.565	489.316	10.693
		*SDRL*	47.773	124.340	462.330	10.715	48.032	136.661	505.755	10.463
	(0.75,0.75)	*ARL*	1.263	49.289	3.037	520.931	1.287	47.693	3.026	520.392
		*SDRL*	0.592	48.517	2.480	504.021	0.619	48.305	2.440	546.134
5	(0.25,0.25)	*ARL*	539.063	6.161	39.520	1.489	471.851	5.297	35.721	1.469
		*SDRL*	558.767	5.820	36.111	0.855	474.843	4.914	36.668	0.853
	(0.25,0.75)	*ARL*	1.540	514.870	6.334	44.574	1.569	518.329	6.770	41.078
		*SDRL*	0.889	517.861	5.557	43.199	0.932	510.305	6.525	39.602
	(0.75,0.25)	*ARL*	37.020	89.434	483.471	6.361	30.799	98.287	491.057	6.561
		*SDRL*	37.766	87.667	458.917	5.547	30.385	93.490	486.265	6.103
	(0.75,0.75)	*ARL*	1.151	31.700	2.529	518.740	1.149	36.183	2.568	524.711
		*SDRL*	0.418	31.245	1.993	515.537	0.396	34.827	1.970	518.414
6	(0.25,0.25)	*ARL*	528.873	3.964	26.911	1.197	502.758	3.822	27.398	1.229
		*SDRL*	506.582	3.167	28.571	0.486	506.234	3.325	28.357	0.541
	(0.25,0.75)	*ARL*	1.369	570.097	5.741	32.673	1.349	505.078	5.605	29.440
		*SDRL*	0.741	560.254	4.867	32.519	0.681	472.832	4.898	29.464
	(0.75,0.25)	*ARL*	23.170	64.967	490.714	4.488	22.741	73.182	499.815	4.616
		*SDRL*	22.606	65.311	487.642	4.030	22.323	70.993	487.262	4.072
	(0.75,0.75)	*ARL*	1.076	24.717	2.053	540.885	1.073	24.115	2.002	498.873
		*SDRL*	0.276	23.223	1.452	546.473	0.289	23.155	1.483	497.693

**Table 7:** *ARL*s along with their respective standard deviations for α = 0.01.

*n*	*LGD* Parameters	Percentile of interest								
			*u* = 0.05				*u* = 0.1			
	(*θ*,*p*) ⇓	(*θ*,*p*) ⇒	(0.25,0.25)	(0.25,0.75)	(0.75,0.25)	(0.75,0.75)	(0.25,0.25)	(0.25,0.75)	(0.75,0.25)	(0.75,0.75)
4	(0.25,0.25)	*ARL*	93.963	3.311	14.509	1.316	91.845	3.125	14.048	1.298
		*SDRL*	92.320	2.779	13.633	0.682	91.780	2.494	13.530	0.624
	(0.25,0.75)	*ARL*	1.504	100.804	4.649	13.315	1.539	98.658	4.727	15.698
		*SDRL*	0.854	100.987	4.049	12.811	0.997	98.223	4.105	14.681
	(0.75,0.25)	*ARL*	14.595	28.281	104.184	3.901	13.217	25.171	96.798	3.690
		*SDRL*	13.667	27.843	103.234	3.391	12.444	25.295	99.974	3.038
	(0.75,0.75)	*ARL*	1.121	13.805	2.088	102.540	1.130	14.405	2.076	102.032
		*SDRL*	0.364	13.274	1.492	99.583	0.376	13.972	1.505	103.669
5	(0.25,0.25)	*ARL*	97.642	2.139	10.475	1.111	101.900	2.313	10.827	1.122
		*SDRL*	95.798	1.554	10.316	0.345	96.782	1.749	10.172	0.373
	(0.25,0.75)	*ARL*	1.293	97.338	4.132	11.011	1.301	93.711	4.012	11.605
		*SDRL*	0.601	98.734	3.546	10.823	0.638	96.052	3.631	11.385
	(0.75,0.25)	*ARL*	10.537	21.029	94.421	2.679	10.198	21.601	96.512	2.614
		*SDRL*	9.947	19.665	99.028	2.049	9.626	20.855	103.041	2.146
	(0.75,0.75)	*ARL*	1.072	11.302	1.731	106.211	1.061	11.087	1.728	102.690
		*SDRL*	0.288	11.075	1.047	105.453	0.239	10.676	1.135	98.139
6	(0.25,0.25)	*ARL*	97.802	1.705	8.069	1.045	100.935	1.787	8.163	1.049
		*SDRL*	94.319	1.105	7.453	0.217	99.464	1.178	7.418	0.216
	(0.25,0.75)	*ARL*	1.183	97.186	3.463	9.157	1.215	101.383	3.697	8.741
		*SDRL*	0.460	91.735	2.914	8.638	0.493	105.699	3.073	8.237
	(0.75,0.25)	*ARL*	8.087	17.759	106.398	2.188	8.468	16.615	96.681	2.094
		*SDRL*	7.290	17.678	106.705	1.497	8.047	16.356	86.325	1.509
	(0.75,0.75)	*ARL*	1.029	8.596	1.564	102.006	1.021	8.019	1.487	93.188
		*SDRL*	0.179	8.062	0.923	107.012	0.143	7.440	0.857	89.433

Generally, based on Tables 5–7, changes in the parameter *p* are detected faster compared to the same amount of change in the *θ* parameter. Moreover, if both *θ* and *p* parameters change, this change is detected much faster. For example, in the first row of Table 5, it can be observed that if the parameters of the *LG* distribution change from (*θ* = 0.25,*p* = 0.25) to (*θ* = 0.75,*p* = 0.75), this change for *u* = 0.05 is detected after approximately 1.91 sampling iterations with an estimated standard deviation of 1.291, and for *u* = 0.1 after approximately 1.884 sampling iterations with an estimated standard deviation of 1.192, using the proposed bootstrap control chart. These demonstrate the very good performance of the suggested bootstrap control charts. Increasing the sample size (*n*) also leads to a reduction in the *ARL*. This means that with an increase in sample size, the bootstrap percentile control chart detects changes in the quality characteristic distribution parameters more quickly. Finally, the choice of percentile *u* (whether 0.05 or 0.1) does not have a significant impact on the performance of the control charts introduced.

In the topic of reliability, there is a well-known proverb that states: "A chain is only as strong as its weakest link." Therefore, in this context, lower percentiles are very important. The upper percentiles of the qualitative distribution are also noteworthy in the quality control field. Consequently, in this section, we have examined our control chart concerning the upper percentages of the *LG* distribution. For this purpose, we considered the two upper percentiles, *u* = 0.75 and *u* = 0.9. Then using the same parameters *p*, θ, and *n* that we applied for the lower percentiles, we calculated the values of *MCL*,*MUCL*,*SDLCL*,*SDUCL*,*ARL*, and *SDRL*. Since the usual level of significance in the field of quality control is *α* = 0.0027, and including other *α* levels would increase the volume of the article without affecting the results, we summarize the calculations in this section in this level only. The results are presented in Tables 8 and 9.

**Table 8:** Average control limits along with respective standard deviations for *α* = 0.0027 with upper percentiles.

*n*	*LGD* Parameters		Percentile of interest							
			*u* = 0.75				*u* = 0.9			
	*θ*	*p*	*MLCL*	*SDLCL*	*MUCL*	*SDUCL*	*MLCL*	*SDLCL*	*MUCL*	*SDUCL*
4	0.25	0.25	1.396704	0.083809	22.90451	0.522355	2.471229	0.136578	34.08251	0.718178
	0.25	0.75	0.291542	0.024741	7.195182	0.164237	0.548673	0.047699	11.03910	0.252600
	0.75	0.25	0.579690	0.058589	16.67074	0.443219	1.085769	0.078979	25.67942	0.564353
	0.75	0.75	0.112470	0.009727	5.185386	0.142526	0.230623	0.014702	8.196925	0.234654
5	0.25	0.25	1.776772	0.111868	21.0075	0.497468	3.111762	0.129332	31.63149	0.662944
	0.25	0.75	0.403769	0.024907	6.611305	0.154807	0.760248	0.047234	10.11829	0.240046
	0.75	0.25	0.780101	0.046061	14.97933	0.340357	1.406237	0.088622	23.42109	0.623889
	0.75	0.75	0.151913	0.008324	4.666191	0.129636	0.281537	0.022554	7.496549	0.229242
6	0.25	0.25	2.187616	0.111572	19.59969	0.432153	3.662271	0.145080	29.93076	0.516982
	0.25	0.75	0.499912	0.027689	6.19028	0.128394	0.914767	0.053117	9.548333	0.264622
	0.75	0.25	0.985795	0.045173	13.87369	0.382528	1.731191	0.073543	21.61003	0.612887
	0.75	0.75	0.192442	0.013194	4.230239	0.115801	0.358699	0.020354	6.898672	0.203837

**Table 9:** *ARL*s along with their respective standard deviations for *α* = 0.0027 with upper percentiles.

*n*	*LGD* Parameters	Percentile of interest								
			*u* = 0.75				*u* = 0.9			
	(*θ*,*p*) ⇓	(*θ*,*p*) ⇒	(0.25, 0.25)	(0.25, 0.75)	(0.75, 0.25)	(0.75, 0.75)	(0.25, 0.25)	(0.25, 0.75)	(0.75, 0.25)	(0.75, 0.75)
4	(0.25,0.25)	*ARL*	379.028	5.755	37.315	1.554	352.225	4.718	30.05	1.533
		*SDRL*	390.830	5.242	37.524	0.971	354.206	4.321	30.493	0.934
	(0.25,0.75)	*MRL*	1.705	357.474	6.025	37.652	1.69	360.7	5.521	34.207
		*SDRL*	1.041	373.675	5.350	38.240	1.152	344.745	4.862	33.638
	(0.75,0.25)	*ARL*	38.54	81.725	365.96	6.413	45.394	62.238	353.409	5.675
		*SDRL*	38.102	79.988	368.051	6.018	44.701	59.997	348.135	4.873
	(0.75,0.75)	*MRL*	1.216	39.765	2.583	377.662	1.201	47.947	2.587	379.736
		*SDRL*	0.536	37.279	1.934	373.009	0.496	46.861	2.012	386.053
5	(0.25,0.25)	*ARL*	398.102	3.721	28.217	1.261	367.691	3.085	22.033	1.245
		*SDRL*	379.817	3.200	27.870	0.572	359.936	2.523	21.016	0.554
	(0.25,0.75)	*MRL*	1.402	358.053	5.075	24.409	1.396	361.772	4.546	20.949
		*SDRL*	0.777	354.159	4.483	22.572	0.722	356.53	4.030	21.293
	(0.75,0.25)	*ARL*	28.728	54.746	365.239	4.141	34.701	49.829	389.714	3.978
		*SDRL*	29.164	52.619	361.055	3.548	35.917	47.512	373.294	3.393
	(0.75,0.75)	*MRL*	1.098	31.663	2.13	348.931	1.114	38.192	2.186	392.606
		*SDRL*	0.320	31.171	1.524	344.112	0.356	37.653	1.596	405.322
6	(0.25,0.25)	*ARL*	379.591	2.294	18.353	1.123	389.071	2.171	17.213	1.123
		*SDRL*	370.477	1.613	17.429	0.382	365.989	1.569	15.628	0.379
	(0.25,0.75)	*MRL*	1.284	379.699	4.231	18.024	1.281	346.931	3.68	17.361
		*SDRL*	0.581	371.708	3.670	17.941	0.578	355.994	3.124	16.431
	(0.75,0.25)	*ARL*	21.533	36.769	340.878	2.892	24.633	32.069	371.14	3.076
		*SDRL*	21.298	36.804	331.754	2.469	24.839	33.532	392.222	2.502
	(0.75,0.75)	*MRL*	1.042	21.425	1.814	351.925	1.052	27.264	1.798	380.687
		*SDRL*	0.205	21.405	1.250	344.355	0.230	26.849	1.183	384.943

The results are quite similar to those obtained for the lower percentiles. Again, the in-control *ARL*s are around 370. Changes in the parameter *p* are detected more quickly compared to the same magnitude of change in the *θ* parameter. When both *θ* and *p* parameters change, this change is detected even faster. Additionally, increasing the sample size (*n*) leads to a reduction in the *ARL*.

## 5. Sensitivity analysis

The accurate estimation of *LG* distribution parameters play a fundamental role in the efficiency and implementation of the control charts presented in this article. Incorrect estimation of these parameters may reduce the *ARL* of the control chart, resulting in a delayed discovery of changes in the distribution of the qualitative characteristic. Therefore, in this section, we analyze the sensitivity of these control charts to the *LG* distribution parameters. We also examine the effect of selecting percentiles on the implementation of the control chart. For this purpose, while keeping other parameters constant, we assess the impact of changes in the parameter for which we want to perform sensitivity analysis on the *ARL* of the control chart.

**Fig 2:**
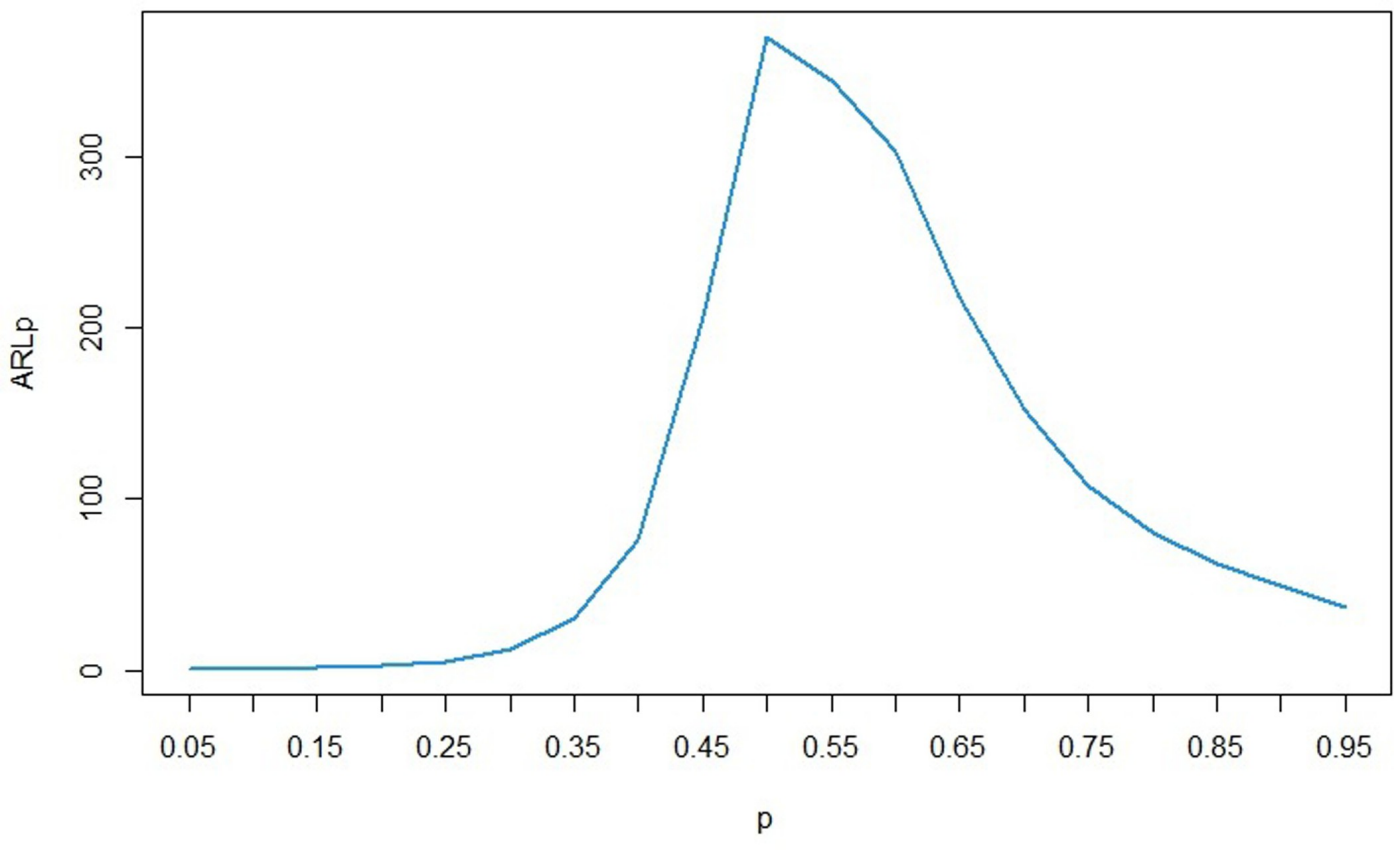
ARL for different values of *p* based on the LGD with (*θ* = 0.5, *p* = 0.05).

**Fig 3:**
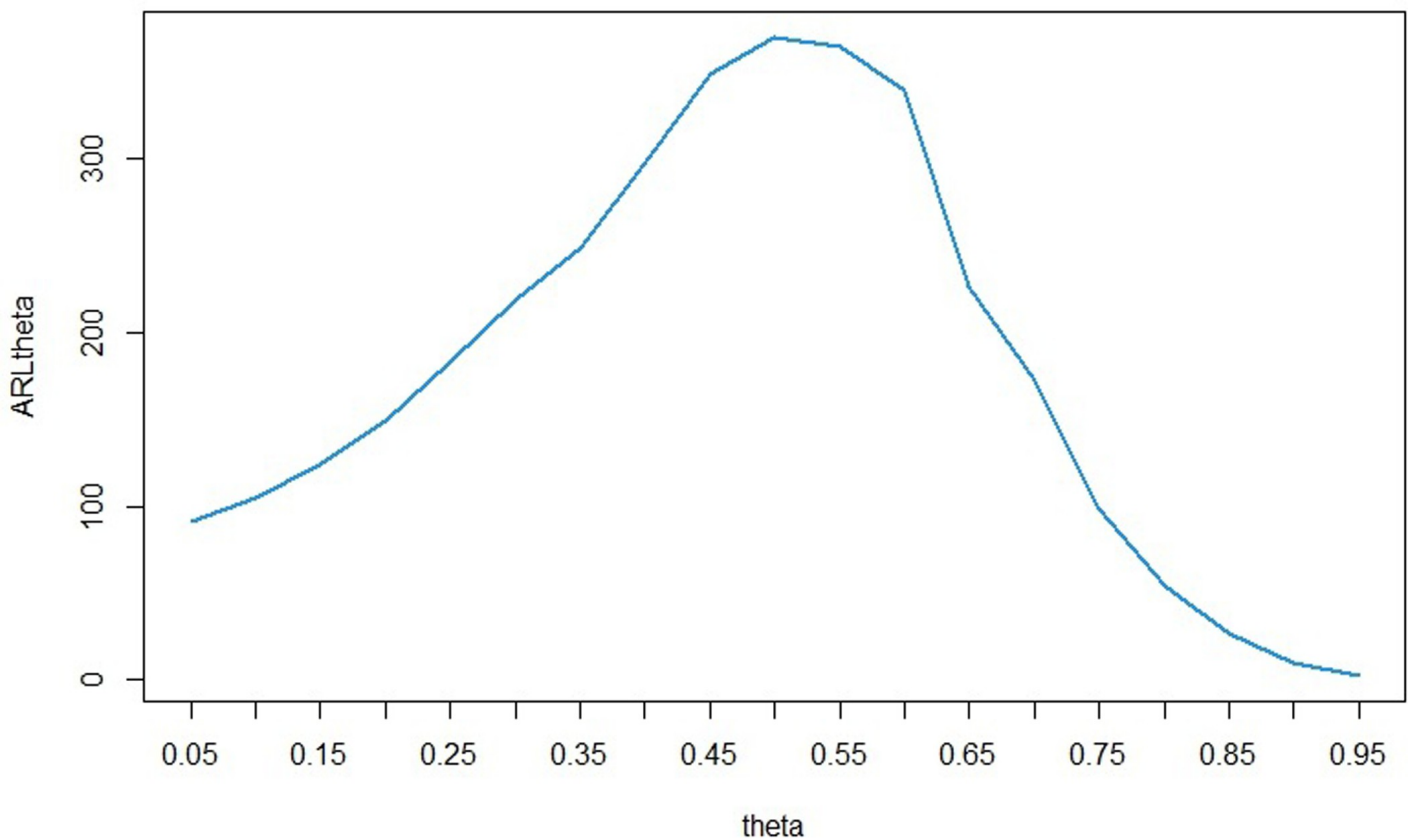
ARL for different values of *θ* based on the LGD with (*θ* = 0.5, *p* = 0.05).

For the calculations in this section, we considered the values of *n* = *5* and *α* = 0.0027, which are commonly used in the field of quality control. Additionally, to analyze the sensitivity of the parameters, we set *u* equal to 0.1. The results remain consistent for other values of *α*, *n*, and *u*. We defined the base distribution as LG with parameters *θ* = 0.5 and *p* = 0.5. Using Algorithm 2, the percentile bootstrap control limits for this distribution were calculated as follows: MLCL = 0.02772982, MUCL = 1.241885, SDLCL = 0.002509196, and SDUCL = 0.03451446. For the sensitivity analysis of the *θ* parameter, we fixed *p* at 0.5 and varied *θ* from 0.05 to 0.95. Conversely, for the sensitivity analysis of the *p* parameter, we fixed *θ* at 0.5 and varied *p* from 0.05 to 0.95. We then assessed the effect of these changes on the *ARL* of the control chart. The results of sensitivity analysis for *p* and *θ* are presented in Figs 2 and 3 respectively.

As seen in Fig 2, the value of *ARL* for *p* = 0.5 (*ARL*_0_) is approximately 370. As we move further away from pp, the *ARL* value (*ARL*_1_) decreases. However, the slope of this decrease is steeper on the left side, such that for equal distances from 0.5, the *ARL* value for lower *p* values is less. For example, for *p* = 0.25, the *ARL* value is approximately 5, while for *p* = 0.75, which is the same distance above 0.5, this value is nearly 108. This demonstrates that a decrease in the *p* parameter is detected by the control chart much earlier than an increase by the same amount in *p*. According to Fig 3, the situation is completely opposite for the *θ* parameter. That is, at equal intervals above and below 0.5, the *ARL* value for upper *θ* values are lower than for lower *θ* values. For example, when the *θ* is equal to 0.75, the ARL value is approximately 99, while this value is approximately 183 for *θ* = 0.25. This means that an increase in the *θ* parameter is detected by the control chart much earlier than a decrease of the same amount in this parameter.

**Fig 4:**
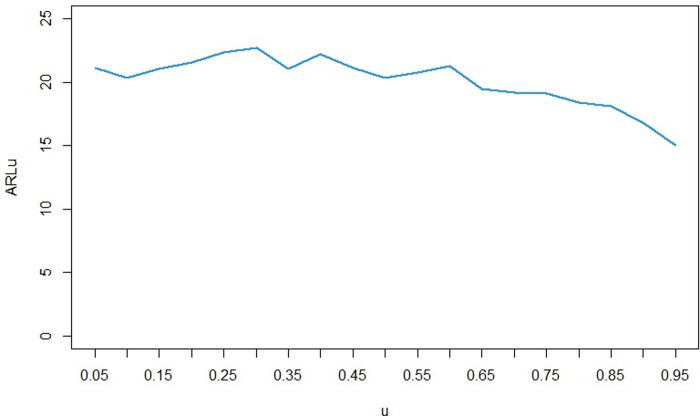
ARL for different values of *u*.

To examine the effect of the selected percentile (*u*) on the implementation of the control chart, we again fixed the value of *n* at 5 and *α* at 0.0027. The base distribution for the qualitative characteristic is considered to be *LG* with *θ* = 0.5 and *p* = 0.5. When this distribution is changed to *LG* with parameters *θ* = 0.75 and *p* = 0.25, we calculated the *ARL* of the control chart for *u* values ranging from 0.05 to 0.95. The results are presented in Fig 4.

According to Fig 4, it can be seen that for each of the 0.05 to 0.65 percentiles, if a change in the *LG* distribution parameters occur as mentioned, this change is discovered on average after 20 samples. However, choosing higher percentiles will detect this change a little earlier. Specifically, the ARL value for *u* = 0.95 is almost equal to 15, which is smaller than that of the other percentiles. Therefore, if we want to detect the change in the *LG* distribution parameters from *θ* = 0.5,*p* = 0.5 to *θ* = 0.75,*p* = 0.25 earlier, it is better to use the 0.95 percentile.

In general, the issue of selecting the appropriate percentile for the bootstrap control chart for the *LG* distribution depends on which parameter changes are most significant to us. Once we determine the extent of increase or decrease in the parameters of interest, we can decide, through simulations similar to this section, which of the percentiles would be more beneficial for the specific issue at hand.

## 6. Illustrative examples

In this section, we analyze the application of the bootstrap percentile control chart for the of the *LG* distribution. This will be demonstrated through a simulated example and a real-world dataset (S1 and S2 Tables).

### 6.1 A simulated example

To create the simulated example in the R software, we initially generated 25 sets of 5 samples each from the *LG* distribution with parameters (*θ* = 0.25, *p* = 0.25) using algorithm 1. Subsequently, with *α* = 0.0027, and following steps 2 to 7 of Algorithm 2, we derived the values for *LCL* and *UCL* for *u* = 0.05 and *u* = 0.1. The average of these values was then used as the *LCL* and *UCL* for phase I. The computed values are as follows: for *u* = 0.05, the *LCL* = 0.06308313 and the *UCL* = 2.079311; and for *u* = 0.1, the *LCL* = 0.1308916 and the *UCL* = 3.448046.

**Fig 5:**
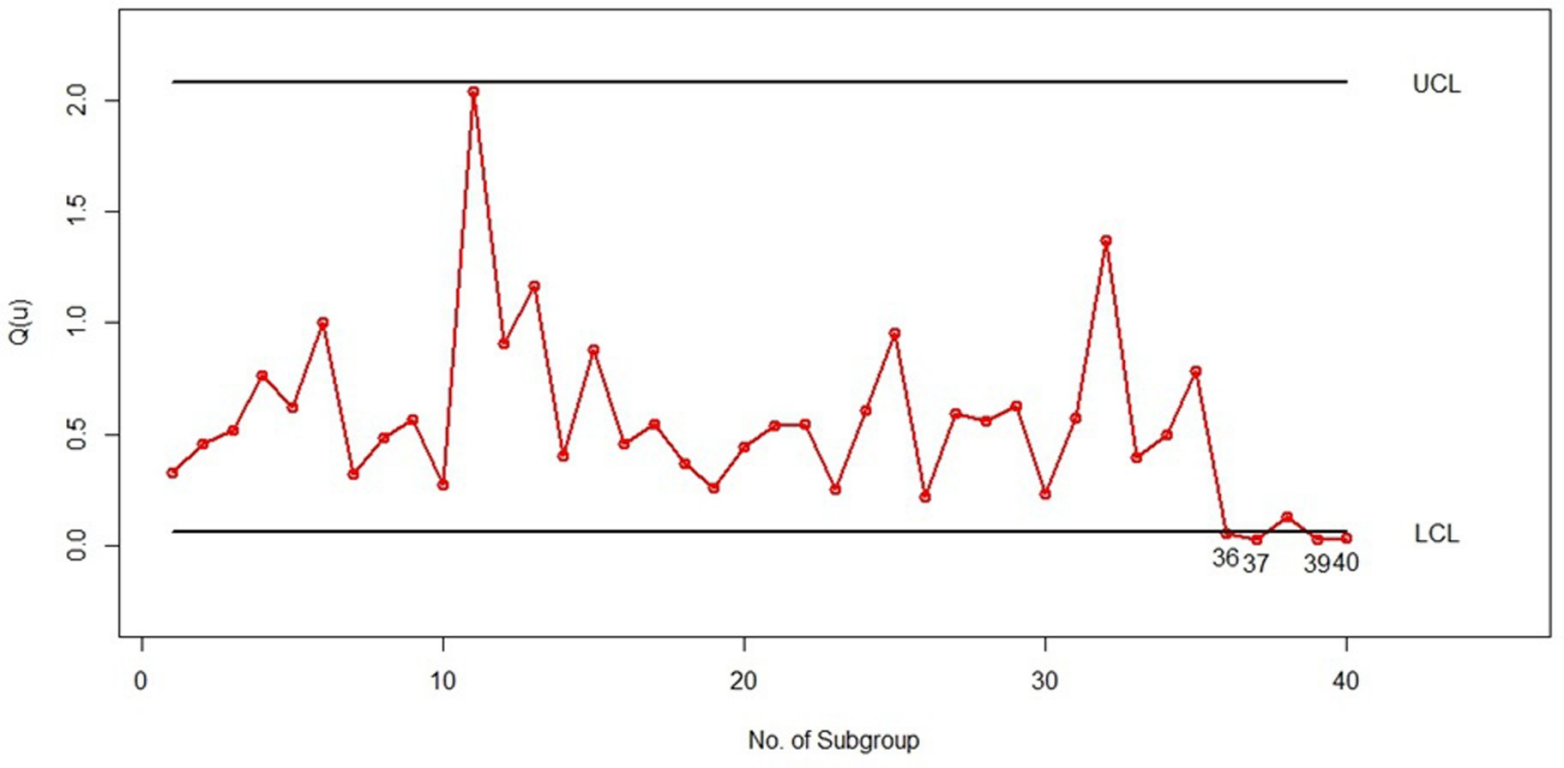
Bootstrap percentile control chart with *u* = 0.5 for randomly generated subgroups of size 5 from the *PLD*.

**Fig 6:**
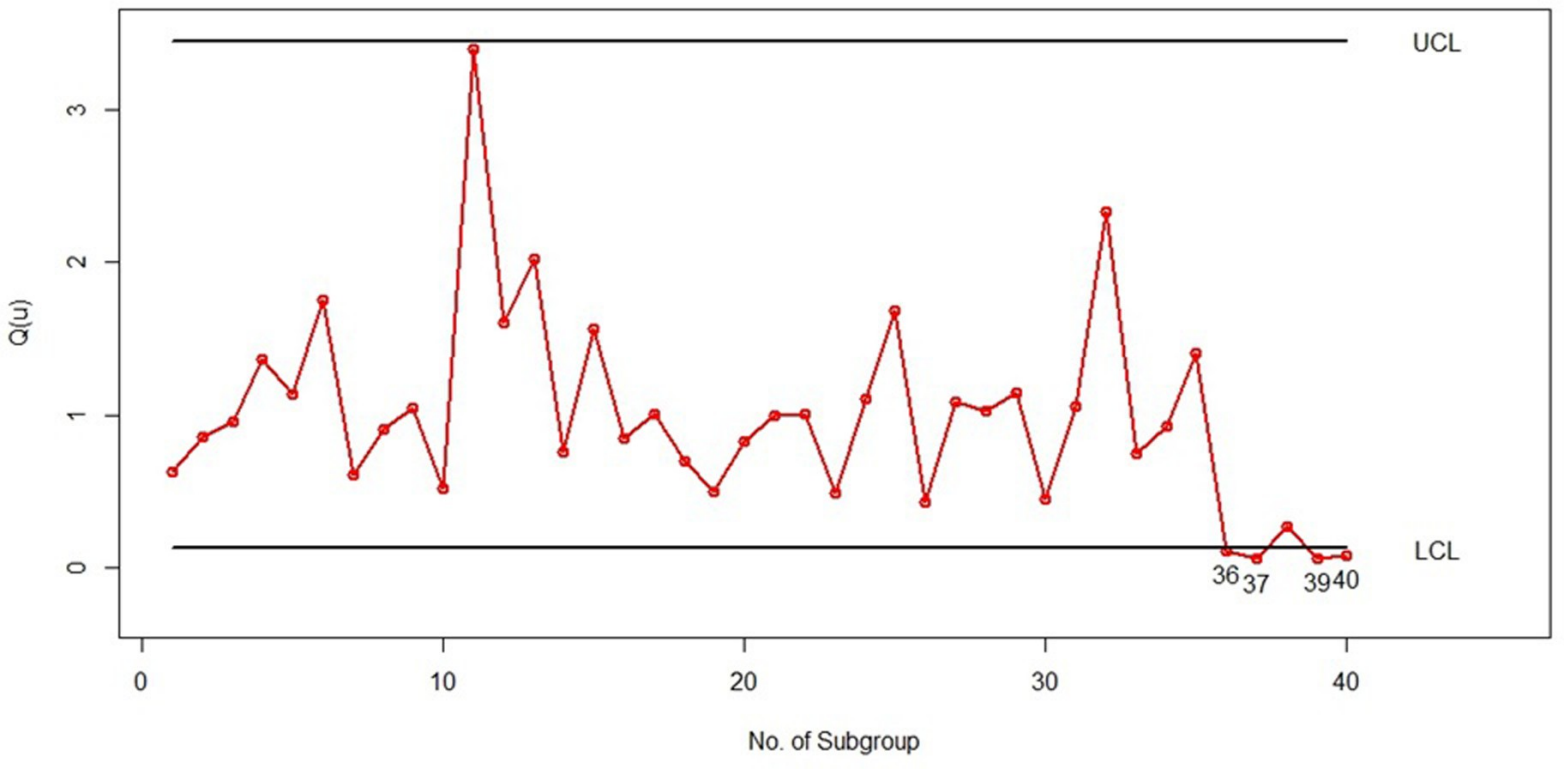
Bootstrap percentile control chart with *u* = 0.1 for randomly generated subgroups of size 5 from the *PLD*.

For the implementation phase (phase II), we initially generated 10 subgroups of size 5 from the *LGD* with parameters (*θ* = 0.25,*p* = 0.25) to demonstrate samples under control (samples 26 to 35). Additionally, we created five subgroups of size 5 from the *LGD* with parameters (*θ* = 0.75,*p* = 0.75) to represent samples out of control (samples 36 to 40). Figs 5 and 6 illustrate the quantile-based bootstrap control charts for the *LGD* subgroups associated with *u* = 0.5 and *u* = 0.1, respectively, covering subgroups 1 to 40 in the simulation. Each point in Figs 5 and 6 represents $\hat{Q}(u)$ calculated from the estimation of the *LGD* parameters ($\hat{\theta }$ and $\hat{p}$) in the corresponding subgroup, which is calculated from Eq 4.

As shown in Figs 5 and 6 it is clear that samples 36, 37, 39, and 40 exhibit out-of-control behavior in both the bootstrap percentile control charts with *u* = 0.05 and *u* = 0.1. It is important to note that the percentiles of process subgroups have shifted starting from sample 36 due to changes in the *LGD* parameters. Both of our control charts effectively detected this shift in sample 36 with an ARL of 1, which is a significant achievement. This scenario demonstrates the strong performance of the bootstrap percentile control charts presented in this article and highlights that the choice of percentile *u* (whether 0.05 or 0.1) has no impact on the performance of the control charts introduced.

**Fig 7:**
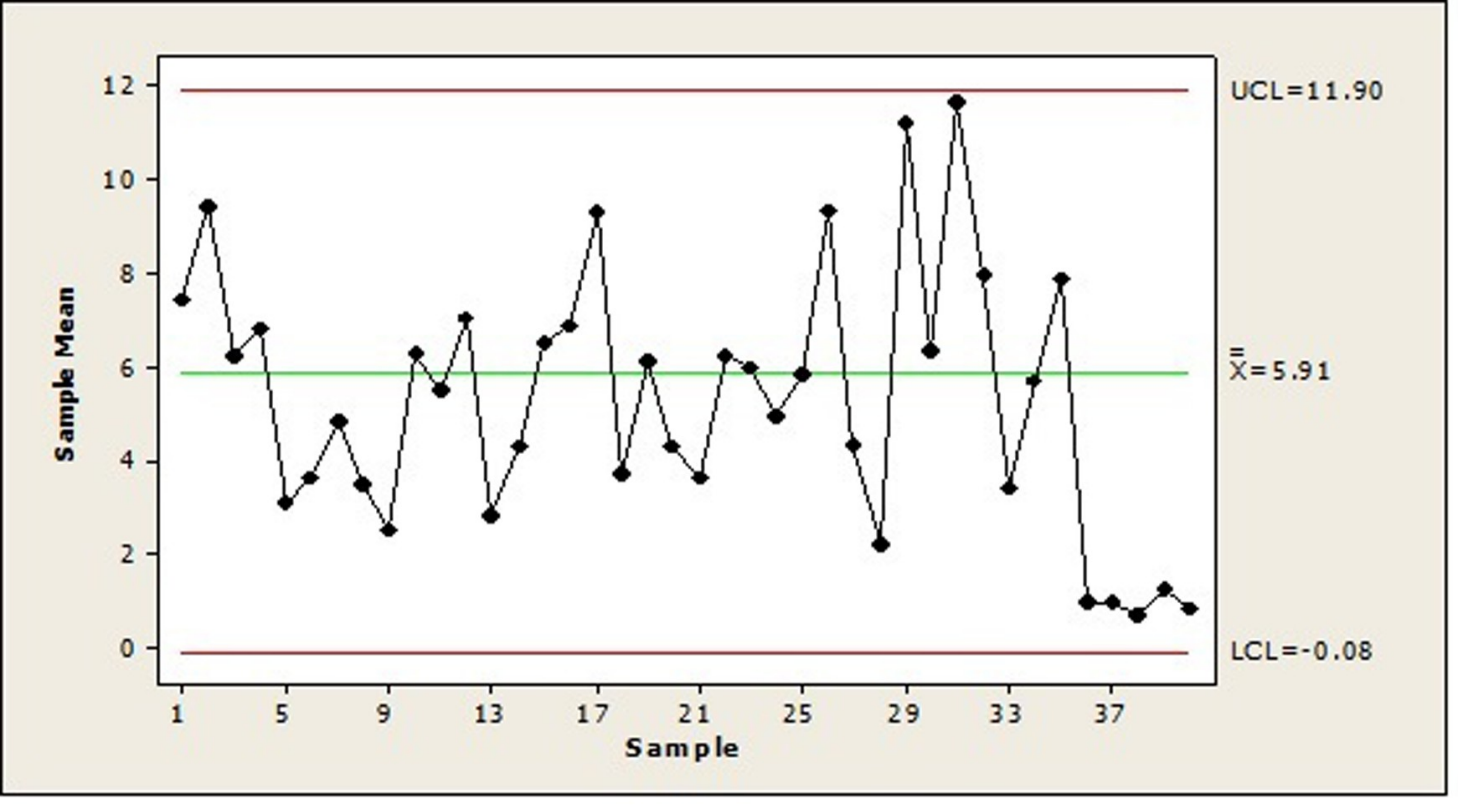
The $\bar{X}$ Shewhart control chart for randomly generated subgroups of size 5 from the *PLD*.

We have also presented the $\bar{X}$ Shewhart control chart for the simulated data in this example, created using Minitab software, as shown in Fig 7. In this control chart, each point represents the average of five subgroups. The lower control limit (*LCL*) and upper control limit (*UCL*) were calculated using the first 25 subgroups as phase 1 data, and the averages of subgroups 26 to 40 were added as phase 2 points. As can be seen in Fig 7, all the points are under control, and this chart could not identify the change in the *LG* distribution parameters from subsamples 36 to 40. Therefore, this example demonstrates that the $\bar{X}$ Shewhart control chart is not suitable for such data. In contrast, according to Figs 5 and 6, our proposed control chart detected this change immediately, indicating its high efficiency.

### 6.2 A real data set example

**Fig 8:**
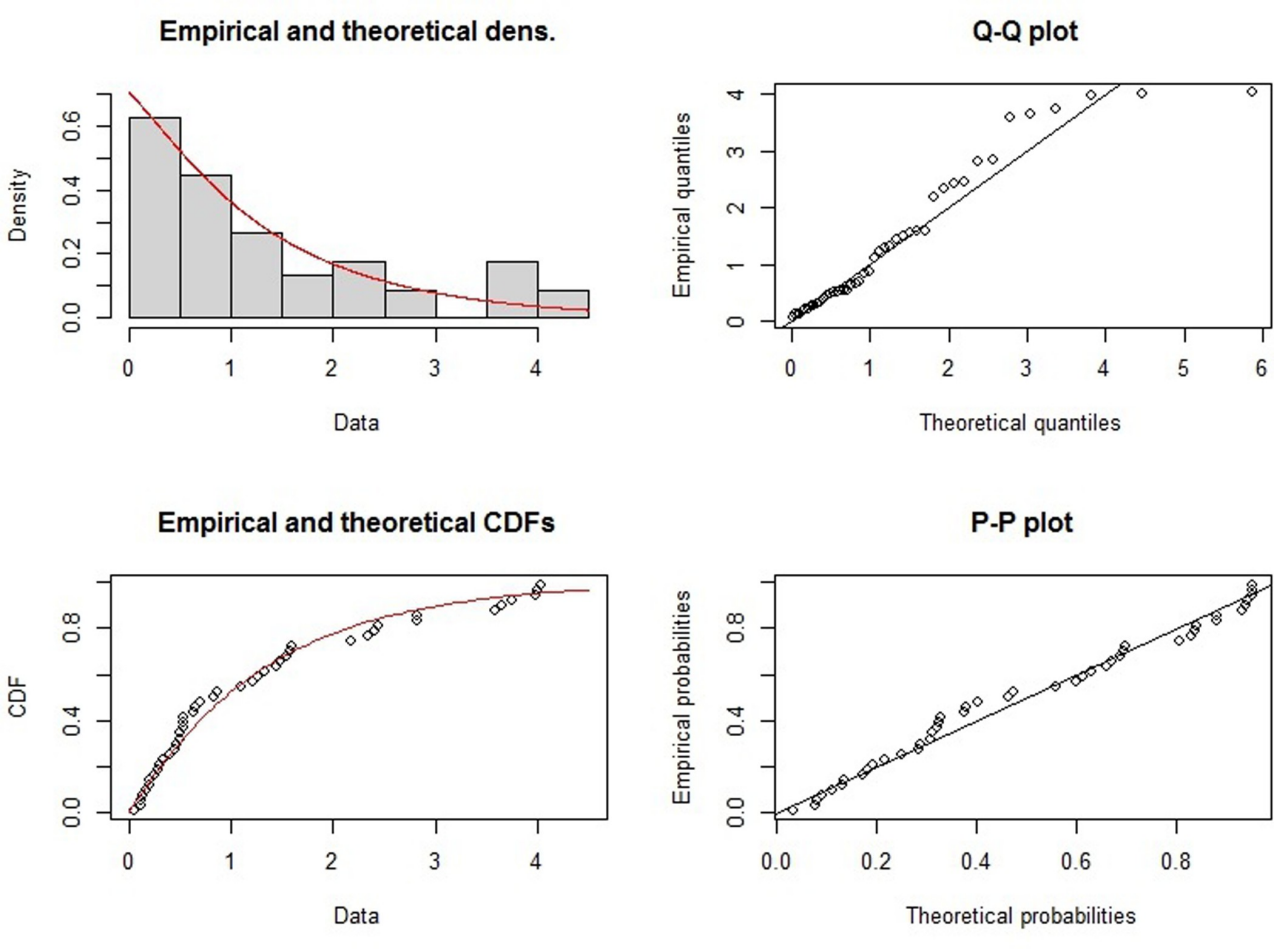
Histogram (top left), Q-Q plot (top right), CDF plot (bottom left), and P-P plot (bottom right) of survival time dataset along with the *LG* distribution fitted to it.

To evaluate the application of the control charts presented in Section 2 to a real dataset, we examined the dataset "survival times in years of gastric cancer patients" obtained from [64]. Given that the data were originally ordered, we randomized them into 9 samples of size 5 to enable their use in the control charts. Table 10 shows the observed subgroups of the dataset. Using the "fitdistrplus" package in R software, we modeled the dataset with the *LG* distributions. The estimated *LGD* parameters were determined as $\hat{\theta }=0.9136358$ and $\hat{p}=0.3791914$ using the maximum likelihood (*ML*) method (stage 2 of Algorithm 2). Additionally, the Kolmogorov-Smirnov test statistic for fitting the *LG* distribution to the dataset is 0.09487884 with a p-value of 0.8124, indicating that the *LGD* fits the dataset well. Furthermore, we calculated the Cramer-von Mises statistic to be 0.06430339, the Anderson-Darling statistic to be 0.46977558, the Akaike Information Criterion (*AIC*) to be 120.345, and the Bayesian Information Criterion (*BIC*) to be 123.9583, once again demonstrating a good fit of the *LGD* to the dataset. Fig 8 shows the histogram, Q-Q plot, CDF plot, and P-P plot of the survival time dataset, along with the *LG* distribution fitted to it.

**Table 10:** Observed subgroups of the survival times dataset, along with the $\hat{Q}$(*u* = 0.05) for each subgroup.

No. of subgroup	Samples in subgroup	$\hat{Q}(u)$
1	1.326, 0.841, 0.282, 2.830, 0.121	0.05837280
2	0.644, 0.197, 1.581, 2.178, 1.553	0.07848288
3	1.447, 2.343, 0.863, 3.658, 0.132	0.11621552
4	4.033, 3.978, 2.416, 0.534, 0.501	0.17214521
5	0.458, 4.003, 0.260, 1.099, 0.696	0.05977463
6	0.164, 1.271, 0.641, 3.743, 0.395	0.05431491
7	0.203, 0.296, 0.529, 1.485, 2.825	0.05447549
8	0.115, 1.589, 2.444, 1.219, 3.578	0.12504756
9	0.540, 0.507, 0.466, 0.047, 0.334	0.02051430

**Fig 9:**
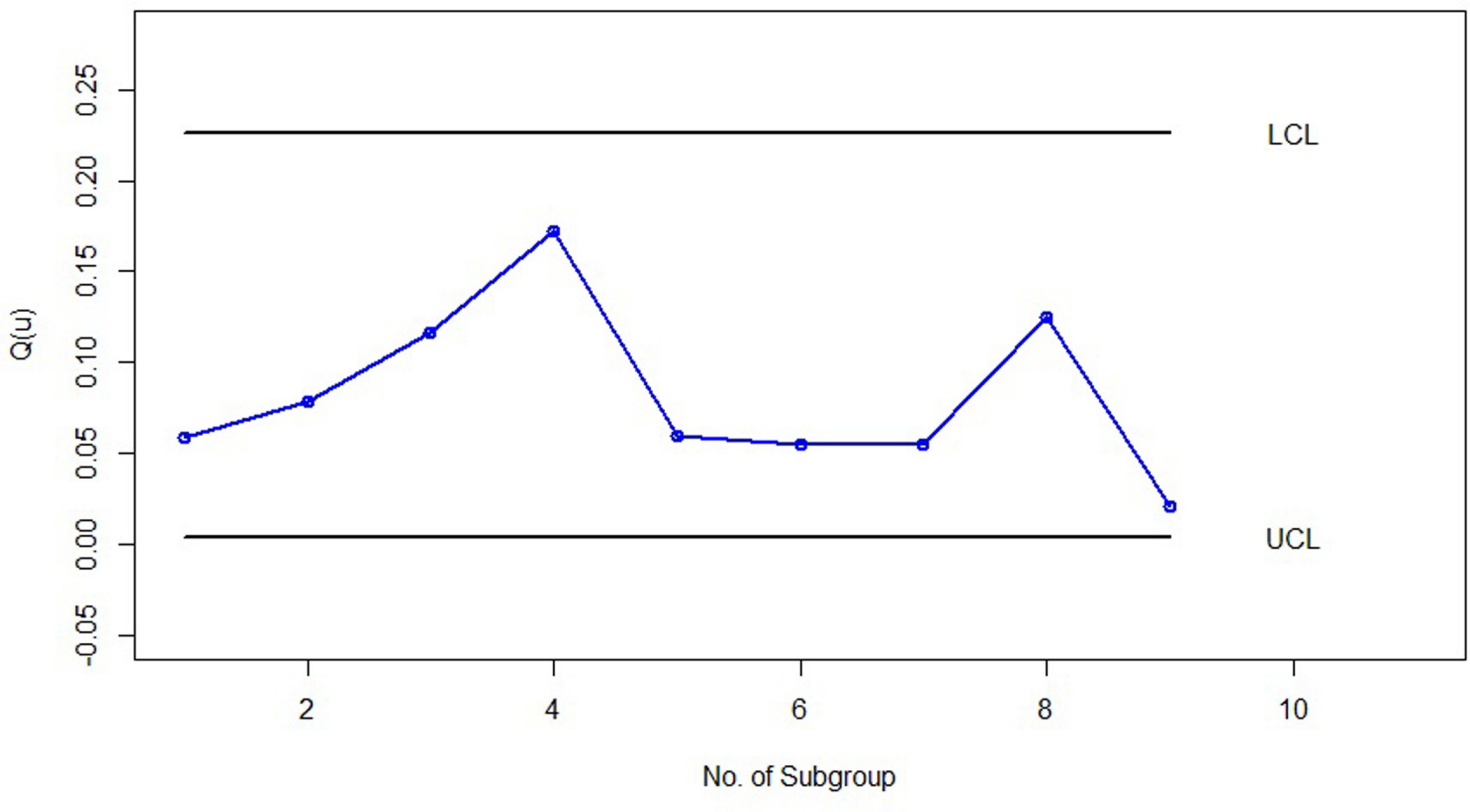
Bootstrap percentile control chart with u = 0.05 for subgroups of size 5 of the survival times data set.

Applying stages 3 to 7 of Algorithm 2 with *α* = 0.0027, *u* = 0.05, *n* = 5, *B* = 10000, and *k* = 100 yielded the following results: *MLCL* = 0.003753354, *SDLCL* = 0.0003751751, *MUCL* = 0.2262595, and *SDUCL* = 0.00220619. Table 10 shows the $\hat{Q}(u)$ for each subgroup, where *u* is set to 0.05, and Fig 9 illustrate the bootstrap percentile control chart for the subgroups of size n = 5 for the survival times in years of gastric cancer patient’s dataset with u = 0.05.

As seen in Fig 9, the 5th percentile of all observations of the subgroups is in control. Therefore, this control chart can be used for phase II monitoring of the 5th percentile subgroups with a sample size of 5 for future data.

### 6.3 A real data set example for phase II

Continuing from the previous example to demonstrate how to implement the control chart introduced for Phase II, we have considered the dataset number 6 presented in [34]. In the mentioned article, the LG distribution was fitted to this dataset with parameters *θ* = 0.5455 and *p* = 0.6348. We want to determine whether we can detect this change in the parameters of the LG distribution using the control chart presented in Phase I for the survival time dataset. For this purpose, we have taken the initial 30 data points of dataset number 6 from [34] and considered them as phase II data in subgroups of 5 from subgroups 10 to 15. Table 11 shows the subgroups along with $\hat{Q}(u)$ corresponding to each subgroup for u = 0.05.

**Table 11:** Observed subgroups of the phase II dataset, along with the $\hat{Q}$(*u* = 0.05) for each subgroup.

No. of subgroup	Samples in subgroup	$\hat{Q}(u)$
10	5.1, 1.2, 1.3, 0.6, 0.5	0.09515162
11	2.4, 0.5, 1.1, 8.0, 0.8	0.12155543
12	0.4, 0.6, 0.9, 0.4, 2.0	0.05121933
13	0.5, 5.3, 3.2, 2.7, 2.9	0.23685374
14	2.5, 2.3, 1.0, 0.2, 0.1	0.06896004
15	0.1, 1.8, 0.9, 2.0, 4.0	0.12247644

**Fig 10:**
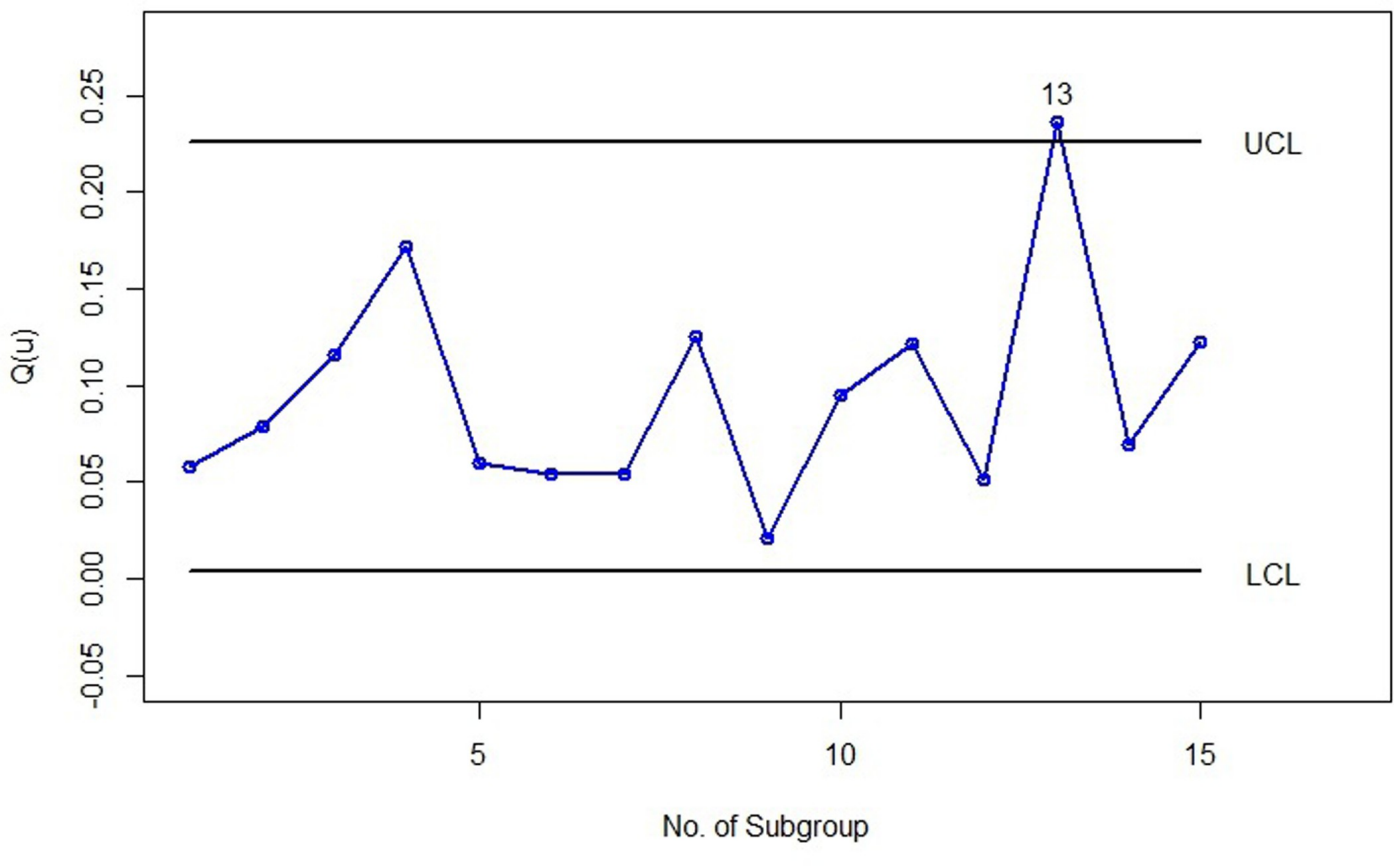
Bootstrap percentile control chart with u = 0.05 for phases I and II data sets.

Fig 10 shows the bootstrap percentile control chart for the of data from phases I and II simultaneously, where the *LCL* and *UCL* are calculated from the data of phase I, and the points on the chart represent the $\hat{Q}(u)$ calculated for each subgroup. In this figure, points 1 to 9 relate to the phase I dataset, while points 10 to 15 pertain to the phase II dataset. As seen in Fig 10, subsample number 13 is out of control. This indicates that the bootstrap control chart has detected a change in the parameters of the *LG* distribution after four sampling iterations.

**Fig 11:**
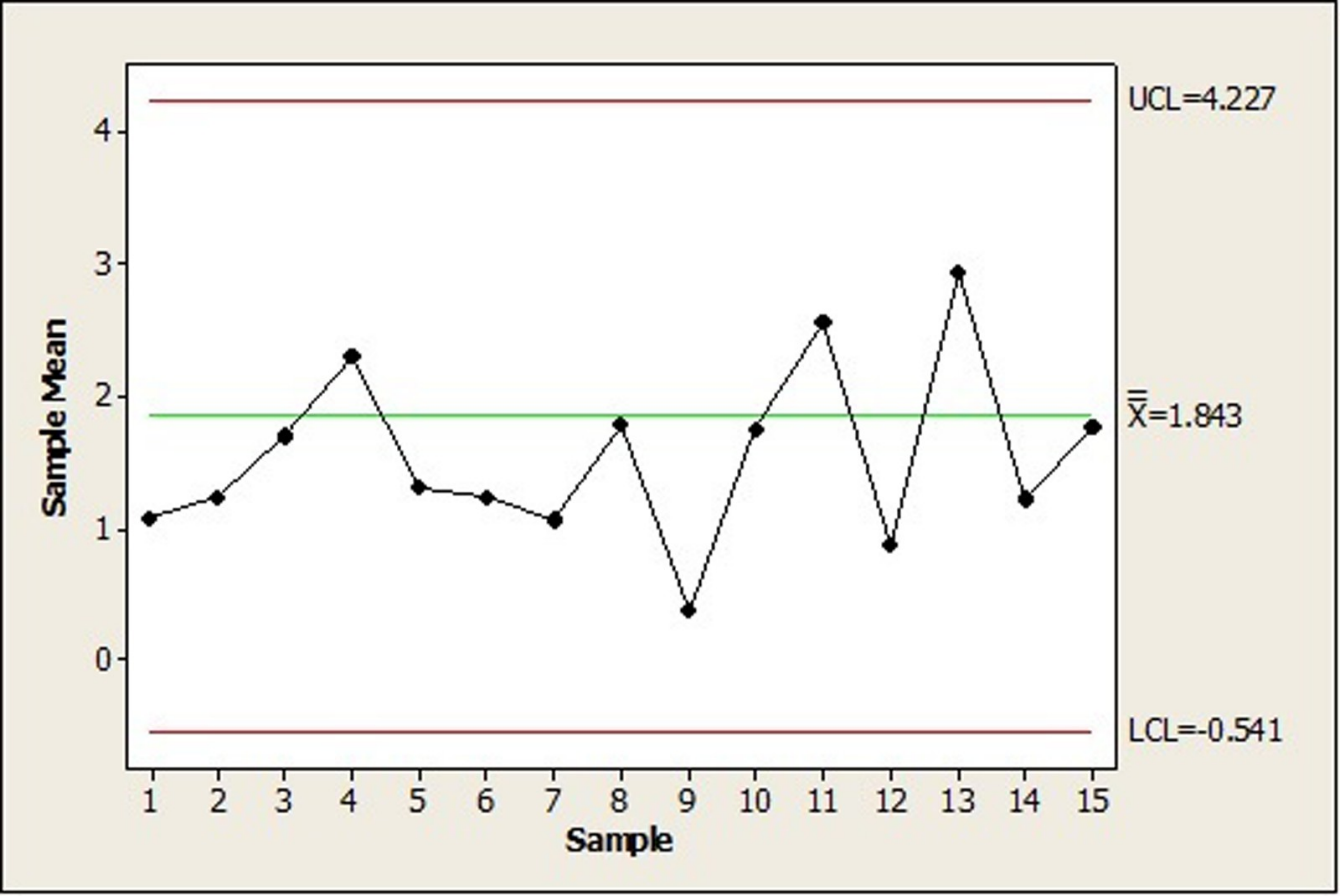
The $\bar{X}$ Shewhart control chart for phases I and II data sets.

Fig 11 shows the $\bar{X}$ Shewhart control chart for the data sets of phases I and II, where the *UCL* and *LCL* are calculated from the phase I data set, and the points on the chart represent the subgroup means. As seen in this figure, all points are under control, and if we were using thiss control chart, we would not be able to detect changes in the parameters of the *LG* distribution.

## 7. Discussion and conclusions

In many industrial settings, quality characteristics often follow skewed distributions, making traditional methods like Shewhart control charts unsuitable. To address this, we can use more flexible distributions, such as the Lindley geometric (*LG*) distribution, which is valuable for modeling material strength and failures. Understanding the strength distribution is crucial for structural design, especially when lower percentiles indicate reduced tensile strength.

This research introduces a new control chart that employs parametric bootstrap techniques for *LG* distribution percentiles. We first outlined key features of the *LG* distribution, including quantile calculation, parameter estimation, and random number generation. Then, we developed an algorithm to determine the upper and lower limits of the bootstrap percentile control chart.

We evaluated the effectiveness of the proposed control chart through simulations, calculating average run lengths (*ARL*s) and their standard deviations in both in-control and out-of-control scenarios. The simulations, conducted using R software, revealed several important findings:

- Larger subgroup sizes result in narrower control limits.
- Higher percentiles lead to wider control limits.
- A lower significance level (*α*) results in wider control limits.
- Increases in *LG* distribution parameters narrow the control limits.
- Changes in the parameter *p* are detected more quickly than changes in the parameter *θ*.
- Detection is faster when both *θ* and *p* are altered.
- Increasing the sample size (*n*) decreases the *ARL*.
- The choice of percentile (0.05, 0.1, 0.75 or 0.95) has minimal impact on the control chart’s performance.

In conclusion, the simulation results demonstrate the high efficiency of the proposed control chart. Its performance was further validated through both simulated and real-world examples, both of which highlighted its exceptional effectiveness.

## 8. Appendix

### 8.1 The R code of Algorithm 1

# Generating random numbers from LGD
library(lamW)
qlingeo<-function(p,Theta, pp)
{
 stopifnot(p < 1, p > 0, Theta> 0)
 x = -lamW::lambertWm1(-(p-1)*(Theta+ 1)*exp(-(Theta+ 1))/(pp*p-1))/Theta- 1/Theta- 1
 return(x)
}
rndlingeo<-function (n,Theta, pp)
{
 y = stats::runif(n, min = 0, max = 1)
 randdata = qlingeo(y, pp, Theta)
 return(randdata)
}

### 8.2 The R code of Algorithm 2

# Constructing the bootstrap percentile control chart for the ***LGD*.**
fit_distribution <- function(sample)
{
y<-sample
logL<-function(x){
n<-length(y)
th<-x[1]
p<-x[2]
lnL<-2*n*log(th)-n*log(th+1)+n*log(1-p)+sum(log(1+y))-th*sum(y)-2*sum(log(1-p*(1+th*y/(th+1))*exp(-th*y)))
return(-lnL)
}
opt<-optim(c(0.5,0.5),lower = c(0.01,0.01),upper = c(10,0.999),logL, method = "L-BFGS-B")
phat <-opt$par[1]
thetahat <-opt$par[2]
(c(opt$par[1],opt$par[2]))
return(c(phat, thetahat))
}
LGBootCC <- function(u, alpha, theta, p, n = 5, BootStrap_iterations = 1E+4,MontiCarlo_iter = 100)
{
LCL <-0
UCL <-0
for(itr in 1:MontiCarlo_iter)
 {
 QS<-0
 Theta<-0
 P<-0
  for(i in 1:BootStrap_iterations)
   {
   sample <- rndlingeo(n, theta, p)
   result <- fit_distribution(sample)
   if (!is.null(result))
   {
    Theta[i] <- result[1]
    P[i] <- result[2]
    QS[i] <- qlingeo(u, Theta[i], P[i])
   }
  }
QS <- sort(QS)
LCL[itr] <- QS[as.integer(BootStrap_iterations * alpha / 2)]
UCL[itr] <- QS[as.integer(BootStrap_iterations * (1 ‐ alpha / 2))]
cat("LCL", LCL[itr], "UCL", UCL[itr], "\n")
}
MLCL <- mean(LCL)
MUCL <- mean(UCL)
SDLCL <- sd(LCL)
SDUCL <- sd(UCL)
cat("Mean of LCL (MLCL): ", MLCL," Mean of UCL (MUCL): ", MUCL, "\n")
cat("Standard deviation of LCL (SDLCL): ", SDLCL," Standard deviation of UCL (SDUCL): ", SDUCL, "\n")
}

### 8.3 The R code of fitting the LG distribution to the survival times dataset

dlingeo<-function (x, pp, Theta)
{
 pdf = (Theta^2/(Theta + 1))*(1 ‐ pp)*(1 + x)*
 (exp(-Theta*x))*(1 ‐ pp*(1 + (Theta*x)/(Theta + 1))*(exp(-Theta*x)))^(-2)
 return(pdf)
}
 plingeo<-function (q, pp, Theta)
{
 cdf = (1 - (1 + (Theta*q)/(Theta + 1))*exp(-Theta*q))/(1 ‐ pp*(1 + (Theta*q)/(Theta + 1))*exp(-Theta*q))
return(cdf)
}
# Run qlingeo function of the Algorithm 1 code
library(fitdistrplus)
servtime<-c(
1.326, 0.841, 0.282, 2.830, 0.121,
0.644, 0.197, 1.581, 2.178, 1.553,
1.447, 2.343, 0.863, 3.658, 0.132,
4.033, 3.978, 2.416, 0.534, 0.501,
0.458, 4.003, 0.260, 1.099, 0.696,
0.164, 1.271, 0.641, 3.743, 0.395,
0.203, 0.296, 0.529, 1.485, 2.825,
0.115, 1.589, 2.444, 1.219, 3.578,
0.540, 0.507, 0.466, 0.047, 0.334)
fitlingeo <- fitdist(servtime,"lingeo", start = list(pp = 0.25,Theta = 0.75),lower = c(0.1,0.2),upper = c(0.99,10))
as.vector(summary(fitlingeo)$estimate)
gofstat(fitlingeo)
plot(fitlingeo)

## Supporting information

S1 Table Observed subgroups of the survival times dataset. (DOCX)

S2 Table Observed subgroups of the phase II dataset. (DOCX)
