# Supplementary material for: A parametric bootstrap control chart for Lindley Geometric percentiles
Source: PLoS One. 2025 Feb 6;20(2):e0316449. doi: 10.1371/journal.pone.0316449 (PMC11801588; doi:10.1371/journal.pone.0316449)
Supplement: S1 Table — (DOCX) [file pone.0316449.s001.docx]

**S1 Table. observed subgroups of the** **survival times dataset**

| **No. of subgroup** | **Samples in subgroup** |
| --- | --- |
| 1 | 1.326, 0.841, 0.282, 2.830, 0.121 |
| 2 | 0.644, 0.197, 1.581, 2.178, 1.553 |
| 3 | 1.447, 2.343, 0.863, 3.658, 0.132 |
| 4 | 4.033, 3.978, 2.416, 0.534, 0.501 |
| 5 | 0.458, 4.003, 0.260, 1.099, 0.696 |
| 6 | 0.164, 1.271, 0.641, 3.743, 0.395 |
| 7 | 0.203, 0.296, 0.529, 1.485, 2.825 |
| 8 | 0.115, 1.589, 2.444, 1.219, 3.578 |
| 9 | 0.540, 0.507, 0.466, 0.047, 0.334 |
