# Supplementary material for: A parametric bootstrap control chart for Lindley Geometric percentiles
Source: PLoS One. 2025 Feb 6;20(2):e0316449. doi: 10.1371/journal.pone.0316449 (PMC11801588; doi:10.1371/journal.pone.0316449)
Supplement: S2 Table — (DOCX) [file pone.0316449.s002.docx]

**S2 Table. observed subgroups of the** **phase II dataset**

| **No. of subgroup** | **Samples in subgroup** |
| --- | --- |
| 10 | 5.1, 1.2, 1.3, 0.6, 0.5 |
| 11 | 2.4, 0.5, 1.1, 8.0, 0.8 |
| 12 | 0.4, 0.6, 0.9, 0.4, 2.0 |
| 13 | 0.5, 5.3, 3.2, 2.7, 2.9 |
| 14 | 2.5, 2.3, 1.0, 0.2, 0.1 |
| 15 | 0.1, 1.8, 0.9, 2.0, 4.0 |
